# Cooperative Dinuclear Activation of a Formate Intermediate in the Hydrogenation of CO_2_ to Methanol

**DOI:** 10.3390/molecules31122047

**Published:** 2026-06-11

**Authors:** Giorgia Gherardini, Simon Mathew, Bas de Bruin, Joost N. H. Reek

**Affiliations:** Homogeneous, Supramolecular and Bio-Inspired Catalysis, Van’t Hoff Institute for Molecular Sciences, University of Amsterdam, Science Park 904, 1098 XH Amsterdam, The Netherlands; g.gherardini@uva.nl (G.G.); s.mathew@uva.nl (S.M.)

**Keywords:** homogeneous catalysis, CO_2_ hydrogenation, methanol synthesis, bimetallic catalysis, formate activation

## Abstract

CO_2_ hydrogenation to methanol is achieved by homogeneous catalysts through a formic acid derivative. Previous studies have focused on using large amounts of additives to activate this intermediate, such as strong acids, amines and alcohols. Hydrogenation of CO_2_ under basic conditions has been reported to only produce highly stable formate salts. We present in this contribution a novel method for formate activation that allows for CO_2_ hydrogenation to methanol under basic conditions, by bimetallic activation of the formate salt by a cobalt and a nickel complex. From various catalytic and stoichiometric experiments, we propose that the nickel catalyst binds the in situ-generated formate to activate it for intramolecular cobalt hydride transfer, leading to an intermediate that can be further hydrogenated to methanol. This strategy could open new avenues in CO_2_ hydrogenation under basic conditions, with implications for both homogeneously and heterogeneously catalyzed processes.

## 1. Introduction

Ample research has been focusing on methods to use CO_2_ as a feedstock for the production of chemicals, both in the context of reducing CO_2_ emissions as well as finding new C1 carbon feedstocks for the future. Such strategies require the combination of CO_2_ capture from the air and synthetic method development, but when successful, this approach conveniently transforms CO_2_ from a pollutant to an economically valuable feedstock [1,2,3,4,5,6,7,8]. The conversion of CO_2_ to methanol is particularly desirable, as CH_3_OH is a stable and highly versatile chemical, which can additionally be used as a liquid organic hydrogen carrier, with a 12.6 wt% of hydrogen content [9,10,11,12,13,14,15,16,17,18,19,20,21,22].

The conversion of CO_2_ into MeOH is currently achieved in industry using heterogeneous Cu/ZnO-based catalysts on Al_2_O_3_ support [23,24]. Despite reaching high TONs, these catalysts present two main limitations: (1) the high temperatures necessary for these catalysts to be active (200–250 °C), and (2) the lack of tuning possibilities for their activity and selectivity. On the other hand, homogeneous catalysts in principle offer great advantages in both of these matters, as they generally work at much lower temperatures and present several opportunities to tune the activity and selectivity of the catalysts.

**Scheme 1:**
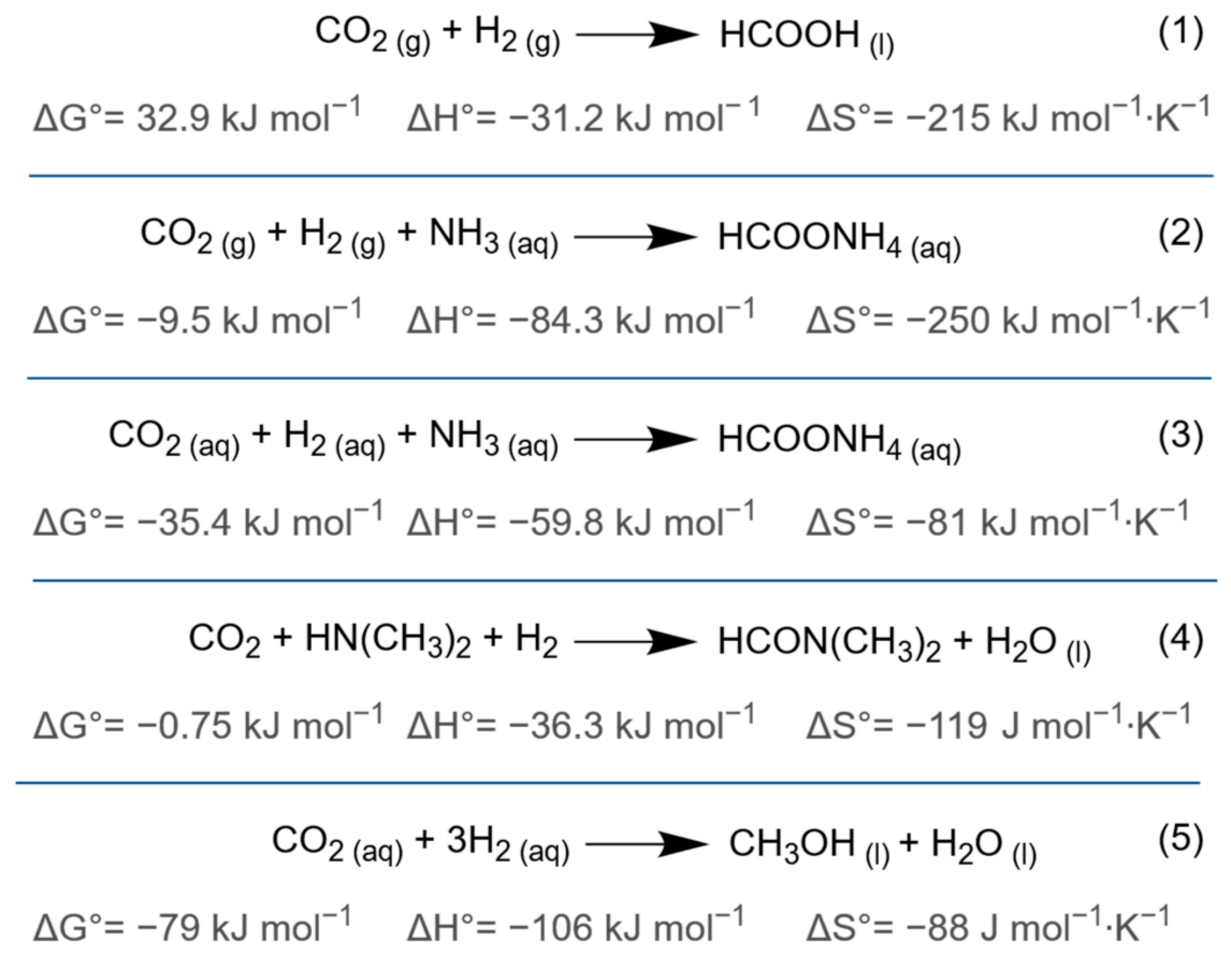
Thermodynamic data for the hydrogenation of CO_2_. ΔG°, ΔH° and ΔS° are calculated at 1 bar and 298.15 K [25,26].

To hydrogenate CO_2_ to methanol, three equivalents of H_2_ are required, and the reaction proceeds usually via a formic acid derivative and formaldehyde as intermediates. The thermodynamics of the reaction dictate that the first hydrogenation step from CO_2_ to formic acid is thermodynamically unfavorable in the gaseous phase (positive Gibbs free energy of formation ΔG°, see Scheme 1, Equation (1)). In the presence of NH_3_ as a base, the reaction becomes thermodynamically feasible because of the formation of a formate salt (Scheme 1, Equation (2)). The thermodynamics of the same reaction in water using dissolved gases at 1 bar make the reaction more favorable, with the ΔG° of the formate salt becoming substantially more negative (Scheme 1, Equation (3)). The reaction of CO_2_ and dihydrogen in the presence of a secondary amine as a protic base leads to the formation of formamide (Scheme 1, Equation (4)), which is less stable than the formation of the formate salt (Scheme 1, Equation (3)), yet more favorable than the formation of formic acid (Scheme 1, Equation (1)). The complete reaction from CO_2_ to methanol is exergonic at room temperature and ambient pressure, with ΔG°= −79 kJ/mol (Scheme 1, Equation (5)). However, the reaction is kinetically challenged, partly because of the different intermediates that can form, which is why it requires catalysts, additives, higher pressures and temperatures for the reaction to occur.

**Scheme 2:**
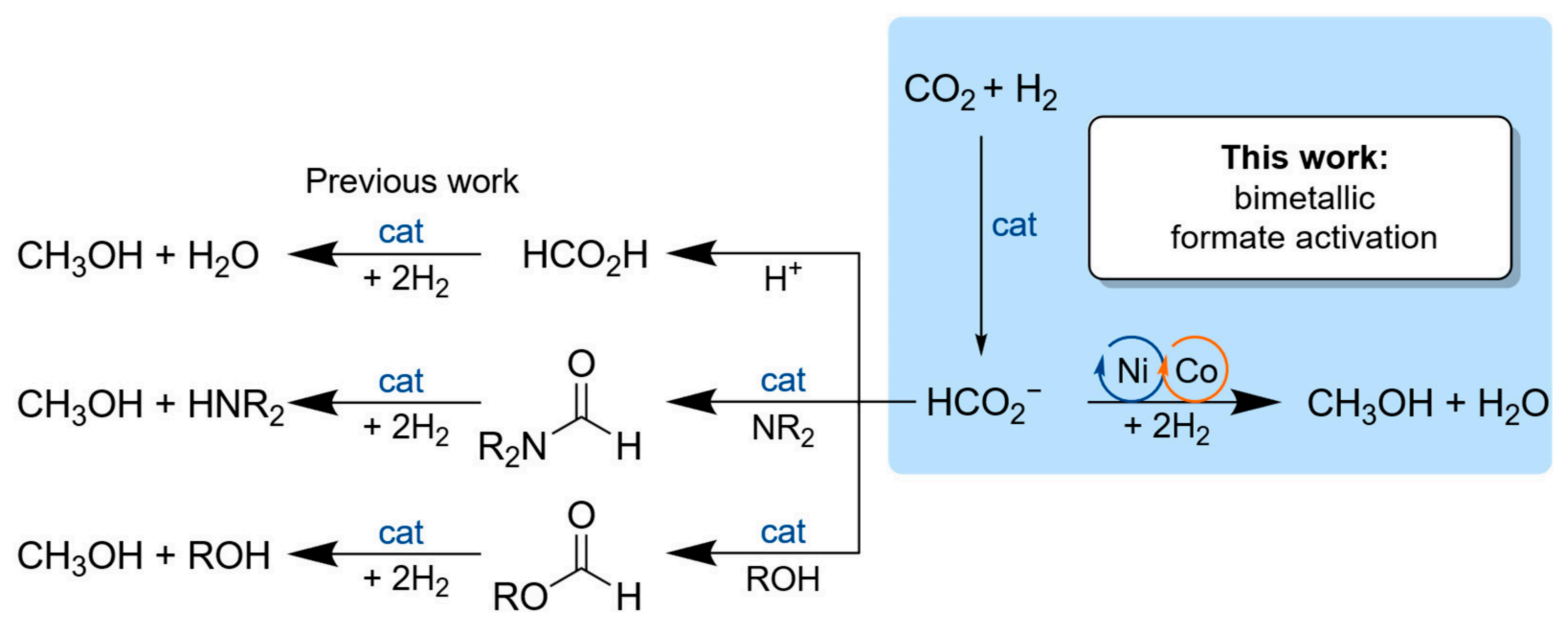
Previously reported strategies for CO_2_ hydrogenation to methanol and their respective formate intermediates.

In particular, the choice of the additive is crucial when targeting the hydrogenation of CO_2_ to CH_3_OH, as it fundamentally determines which type of intermediate will be accessed in the reaction mechanism. Higher-energy intermediates for the first hydrogenation step such as formic acid (Scheme 1, Equation (1)) or formamides (Scheme 1, Equation (4)) yield a more favorable thermodynamic driving force for the second and third hydrogenation steps to produce methanol. This has been widely reported in the literature (Scheme 2). Acid-assisted CO_2_ hydrogenation employing triphos (1,1,1-tris(diphenylphosphinomethyl)ethane)-based metal complexes under highly acidic conditions proceeds via the unstable formic acid intermediate, which is readily hydrogenated to methanediol and subsequently to methanol. Several examples of these systems have been reported, with TONs reaching 2148 using a Ru-based complex in combination with Al(OTf)_3_ for 20h at 140 °C and TONs up to 125 for a Co-based catalyst in combination with HNTf_2_ for 24 h at 100 °C [27,28,29,30,31,32,33]. Previously reported alcohol- and amine-assisted CO_2_ hydrogenations to MeOH obtain, respectively, a formamide and a formate ester, high-energy intermediates that allow for further hydrogenation to MeOH by increasing the electrophilicity of the carbon center [34,35,36,37,38,39]. These systems report TONs up to 9900 using a Ru-aminophosphine-based complex in combination with the polyamine PEHA (pentaethylenehexamine) for 244 h at 145 °C and TONs up to 590 for an Fe–pincer complex in combination with morpholine for 32 h at 100 °C [34,39,40]. Despite their good performance, these systems require a large amount of additive for the reaction to occur. Importantly, achieving CO_2_ hydrogenation under basic conditions is more desirable from an industrial standpoint, as it provides the opportunity of combining the catalytic conversion of CO_2_ with CO_2_-capturing methods, yet this is much more challenging [1,2,6].

Several examples, including cobalt and nickel complexes, have been reported for the hydrogenation of CO_2_ to a formate salt or a formamide derivative under basic conditions, although without forming methanol as a result of the general stability of these salts (Scheme 1, Equation (3)) [41,42,43,44]. Hydrogenation of the more stable but more easily accessible free formate intermediate to methanol has, to the extent of our knowledge, never been reported [36,37,38,39]. In this contribution, we report a catalytic system that combines a novel cobalt-based water-soluble complex and the commercially available [Ni(acac)_2_] for the hydrogenation of CO_2_ to methanol under basic conditions (Scheme 2). In this system, the nickel center activates the in situ-formed formate intermediate to further react with the Co complex for the formation of the methanol product. To the best of our knowledge, this is the first example of two catalysts acting cooperatively in the activation of the HCOO^−^ intermediate to achieve methanol production from carbon dioxide under basic conditions.

## 2. Results and Discussion

To perform the hydrogenation of CO_2_ to methanol under basic conditions without the use of a large excess of additive, we hypothesized that the activation of the stable formate intermediate for further hydrogenation required two cooperating metal-based catalysts. Firstly, we synthesized and characterized a cobalt-based precatalyst that would perform the hydrogenation of CO_2_ to formate under basic aqueous conditions. Subsequently, we selected a nickel-based complex as the formate activation catalyst. We then investigated the combination of the two in catalysis for methanol production from CO_2_. Finally, we performed further studies to understand the mechanism of the hydrogenation of the formate salt.

### 2.1. Synthesis and Characterization of Precatalyst [Cp*Co(4DHBP)I]

As the catalyst for the first hydrogenation of CO_2_ to HCOO^−^, we sought a first-row transition metal-based catalyst that could operate under aqueous basic conditions at relatively low temperatures with good activities. Previously reported [Cp*Co^III^(4DHBP)X] (Cp* = 1,2,3,4,5-pentamethylcyclopentadienyl, 4DHBP = 4,4′-dihydroxy-2,2′-bipyridine, X = Cl, H_2_O) complexes by Himeda [45] matched with our initial requirements. By hypothesizing an increased hydricity with a lower oxidation state of the central metal [46] and an increased activity with the softer iodine ligand, we synthesized the water-soluble cobalt-based complex [Cp*Co^II^(4DHBP)I] as the precatalyst for the hydrogenation of CO_2_ to HCOO^−^.

**Figure 1:**
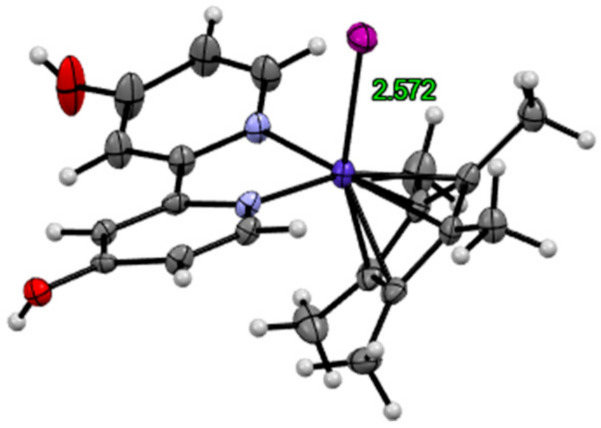
X-ray crystal structure of [Cp*Co(4DHBP)I]. The distance of the Co-I bond is highlighted. Color code: white = hydrogen; grey = carbon; red = oxygen; lilac = nitrogen; purple = cobalt; dark pink = iodine.

[Cp*Co^II^(4DHBP)I] was synthesized by the reaction of precursor [Cp*CoI_2_] with 4DHBP in DCM at 35 °C for 18 h. The complex was isolated as deep-purple air-stable crystals by layering a saturated DCM solution with hexane. The obtained complex is a paramagnetic Co^II^ complex retaining an iodide ligand. Its structure was elucidated by single-crystal X-ray diffraction (Figure 1) and was confirmed to be similar to previously reported [Cp*Co^III^(4DHBP)] complexes by Himeda, though with a few differences [45].

**Scheme 3:**
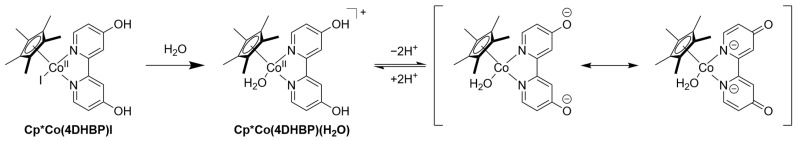
Formation of [Cp*Co(4DHBP)(H_2_O)] from [Cp*Co(4DHBP)I] upon dissolution in H_2_O and double deprotonation of [Cp*Co(4DHBP)(H_2_O)]. The counterion (I^−^ or HCO_3_^−^ from the added base) is omitted for clarity.

Because of the bigger size and softer character of the iodide ligand, the measured Co-I distance in the obtained structure is significantly larger (Figure 1, 2.572 Å) than the previously reported Co-Cl in [Cp*Co^III^(4DHBP)Cl] and Co-O in [Cp*Co^III^(4DHBP)(H_2_O)] complexes (2.297 Å and 1.952 Å, respectively). Iodide can therefore be considered as a quite labile ligand, which is readily lost by dissolving [Cp*Co^II^(4DHBP)I] in H_2_O to form the cationic complex [Cp*Co^II^(4DHBP)(H_2_O)] (Scheme 3).

As previously reported in similar Co^III^-based catalysts, deprotonation of the hydroxide groups (Scheme 3) on the bipyridine backbone leads to an increase in activity due to their better stability and stronger electron-donating ability [45,47]. UV–Vis analysis of the dissolved complex [Cp*Co(4DHBP)(H_2_O)] indicated that at the pH of the catalytic experiments (pH 8.6), the hydroxyl groups on the ligand backbone are indeed deprotonated (Supporting Information, Figures S2 and S3).

### 2.2. Catalytic Activity of Combined [Cp*Co(4DHBP)I] and [Ni(acac)_2_]

We hypothesized the use of a metal complex for the activation of HCOO^−^. Several studies on formato–nickel complexes have detailed the accessible decarboxylation mechanism that these compounds can undergo at ambient pressure, suggesting the activation of the formato ligand when bound to the metal center [48,49,50]. We selected the water-soluble [Ni(acac)_2_] as the possible second catalyst for methanol production, as it presents no additional reactivity towards CO_2_ but can readily bind free formate.

The catalytic performance under various conditions was explored in basic aqueous media under 50 bar (1:4 CO_2_:H_2_) at 100 °C, and product analysis was performed after 24 h of reaction (Table 1). When [Cp*Co(4DHBP)I] or [Ni(acac)_2_] was used as a single catalyst in a batch reaction, the formate salt was the only product obtained, and no catalytic amounts of methanol could be detected under these conditions (entries 1 and 2). In particular, [Cp*Co(4DHBP)I] was observed to catalyze the hydrogenation of CO_2_ to HCOO^−^ with good TONs (entry 1, HCOO^−^ TON 92), indicating [Cp*Co(4DHBP)I] as a promising catalyst to be combined to a formate activation strategy. When a 1:1 mixture of these two catalysts, [Cp*Co(4DHBP)I] and [Ni(acac)_2_], was applied under otherwise similar conditions, the hydrogenation of CO_2_ provided catalytic formation of methanol (entry 4, HCOO^−^ TON 4.2, MeOH TON 4.0). Performing the reaction in the absence of CO_2_ under these conditions afforded no product, confirming that CO_2_ is the source of both formate and methanol (entry 3), and NaHCO_3_ only has the role of base.

Having established that the hydrogenation reaction proceeds to form methanol, some further optimization and control experiments were performed. Increasing the amount of [Ni(acac)_2_] from 3 µmol to 12 µmol resulted in an increase in the TONs to 13.2 for formate and 12.1 for methanol (entry 6). In contrast, increasing the amount of [Cp*Co(4DHBP)I] to 10 µmol did not result in a similar increase in TONs (entry 5, HCOO^−^ TON 1.0, MeOH TON 1.6). It is possible that decomposition of the cobalt complex occurs at higher concentrations, as also evidenced by the formation of a black residue observed at the end of the reaction. When the amount of NaHCO_3_ was decreased from 4 to 2 mmol, the TON to methanol remained roughly the same, while the TON to formate significantly decreased (entry 8, HCOO^−^ TON 1.1, MeOH TON 3.5). This indicates that the base is needed to convert CO_2_ to free formate, in line with the anticipated activation of [Cp*Co(4DHBP)(H_2_O)] by deprotonation (Scheme 3) and the stabilization of HCOO^−^. Interestingly, NaHCO_3_ does not seem to play a role in the subsequent hydrogenation of formate to MeOH.

When [Ni(COD)_2_] was used instead of [Ni(acac)_2_]_,_ an increase in the formation of formate was observed, while a similar methanol production was obtained (entry 7, HCOO^−^ TON 5.9 and CH_3_OH TON 2.9). These results indicate that [Ni^0^(COD)_2_] and [Ni(acac)_2_] can access the same species in the reaction mechanism for the hydrogenation to methanol.

### 2.3. Mechanistic Studies

*Ligand scrambling*: Because the complexation of [Ni(acac)_2_] by bipyridine ligands with different substitution patterns in the presence of a base was previously reported [51], we investigated the possibility of ligand scrambling under the reaction conditions.

ESI–MS analysis of the reaction mixture at the end of a 24 h catalytic reaction revealed the presence of both [Ni(acac)(4DHBP)] (*m*/*z* = 345.039 a.m.u.) and [Ni(4DHBP)_2_] (*m*/*z* = 434.0492 a.m.u.) (Supporting Information, Figure S5). It can be deduced that ligand scrambling happens under the reaction conditions, though it is unclear to what extent this occurs and if it is relevant for the overall hydrogenation reaction from CO_2_ to methanol. We therefore synthesized the “[Ni(4DHBP)_2_]” species and applied it in the hydrogenation of CO_2_ under our otherwise standard conditions. Product analysis at the end of the reaction showed that only the formate product was formed, and methanol could not be detected (Supporting Information, Table S2). This shows that [Ni(4DHBP)_2_] can form under catalytic conditions but that it has no role in the hydrogenation to methanol.

*Formate activation and methanol production*: As previously reported in a computational study by Yang et al. on similar Cp*Co(bipy) complexes at pH 7, the second hydride transfer to formic acid to form methanediol is the rate-determining step of the whole process [47]. This step becomes even more difficult at higher pH levels, as the electrophilicity of the carbon center is lower and the formate salt is stabilized by a base [52]. Therefore, the formate intermediate needs to be activated in order to be further hydrogenated.

**Scheme 4:**
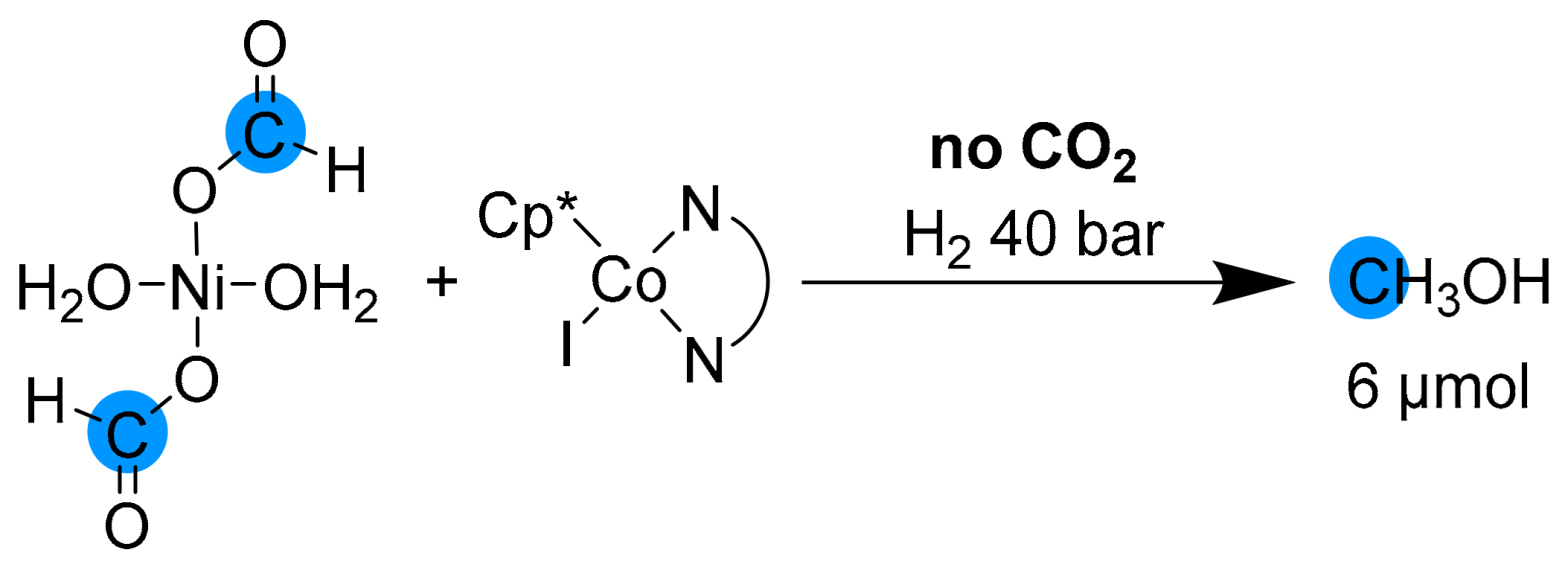
Stoichiometric methanol production by [Cp*Co(4DHBP)I] and Ni(OOCH)_2_·2H_2_O in the absence of CO_2_. Conditions: [Cp*Co(4DHBP)I] 30 µmol, Ni(OOCH)_2_·2H_2_O 30 µmol, NaHCO_3_ 4 mmol, pH_2_ 40 bar, 5 mL H_2_O/THF (4:1), 100 °C, 24 h. CH_3_OH µmol is calculated by GC using 1-butanol as internal standard.

A nickel–formato species is easily formed by coordination of the free formate with a nickel complex, as proven by facile coordination of a formato ligand on [Ni(acac)_2_] and [Ni(4DHBP)_2_] species (Supporting Information, Figures S15–S17) [48,49,50,53]. The possibility of a bimetallic hydride transfer between [Cp*Co(4DHBP)I] and a nickel-bound activated formate species was investigated by using the commercially available salt Ni(OOCH)_2_·2H_2_O combined with [Cp*Co(4DHBP)I] (Scheme 4).

Under H_2_ and CO_2_, both catalysts obtained both formate and methanol catalytically (HCOO^−^ TON 27.5, CH_3_OH TON 6.7), with an increased TON in formate and a similar TON in methanol compared to catalysis with Ni(acac)_2_. In the absence of [Cp*Co(4DHBP)I], no product was obtained, indicating that the cobalt catalyst is necessary for the reaction to occur. In a stoichiometric experiment combining [Cp*Co(4DHBP)I] and Ni(OOCH)_2_·2H_2_O conducted in the absence of CO_2_, a stoichiometric amount of methanol was detected after 24 h (Scheme 4). In this case, the formate ligands on Ni(OOCH)_2_·2H_2_O are the only source of methanol. Gas analysis of the headspace revealed no CO_2_ presence. 

**Scheme 5:**
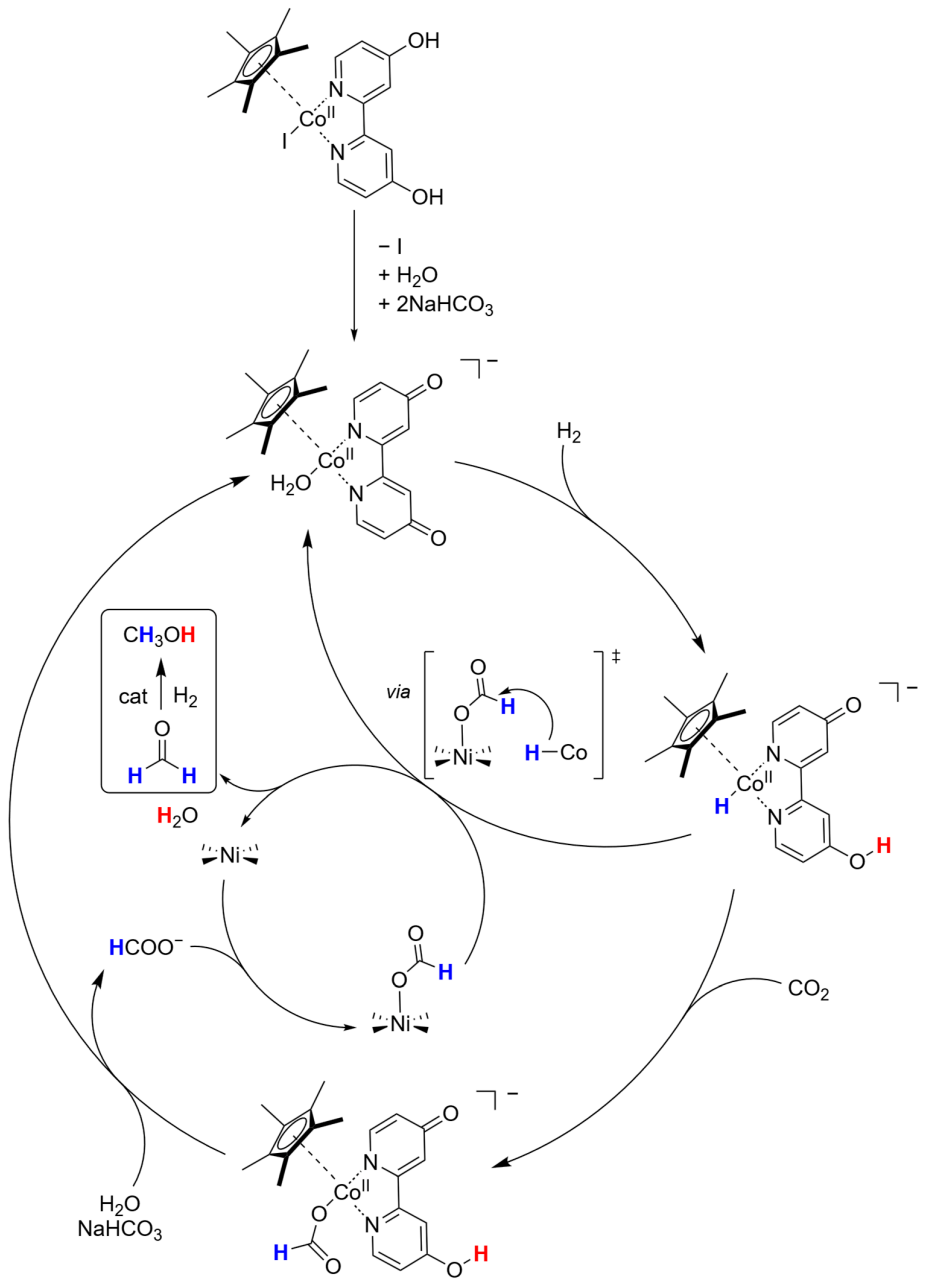
Proposed key hydride transfer between a cobalt hydride and a nickel-bound formate. ‡ = transition state.

Based on this evidence, we propose a mechanism (Scheme 5) in which [Cp*Co(4DHBP)I] readily exchanges the iodide ligand for a water molecule and the ligand 4DHBP backbone is deprotonated by NaHCO_3_. The formation of the Co-H bond is likely facilitated by the protonation of the 4-pyridone unit and the subsequent rearomatization to pyridinol [45]. The reaction of [Cp*Co(4DHBP)H] with CO_2_ affords the formation of a cobalt–formato intermediate, which can regenerate the active catalyst by decoordination of HCOO^−^ (stabilized by the basic environment) and additional deprotonation of the ligand backbone. The formate salt is then activated by binding to the nickel center, and a hydride is transferred to the carbon of this formato species by [Cp*Co(4DHBP)H] through a bimetallic interaction (Scheme 5). Because a catalytic conversion of CO_2_ to methanol was observed when using both [Ni^0^(COD)_2_] and [Ni^II^(acac)_2_], it was deduced that both can access the same active species of unknown oxidation state. The presumably formed methanediol, bound to the nickel center, performs dissociation and dehydration to obtain formaldehyde and a H_2_O molecule. According to various reports, subsequent hydrogenation of formaldehyde to release methanol and a H_2_O molecule is highly favored [17,25,27,54]. Indeed, when [Cp*Co(4DHBP)I] and [Ni(acac)_2_] were tested for formaldehyde hydrogenation, both catalysts obtained methanol both in combination and alone (Supporting Information, Section SII.E).

## 3. Materials and Methods

### 3.1. General Methods

All manipulations were performed with rigorous exclusion of oxygen using standard Schlenk techniques on a dual manifold Schlenk line with N_2_/Ar or a glove box filled with N_2_ unless stated otherwise. All solvents were degassed before use by bubbling Ar through them for an extensive period of time. EtOH was obtained with AcroSeal in 99.9% purity and was used without further purification. DCM was dried over molecular sieves (3 Å). H_2_ DIN 1 and CO_2_ DIN 6 were obtained from Nippon Gases Netherlands (Vlaardingen, The Netherlands). All other reagents were obtained commercially (from Merck, Darmstadt, Germany or TCI Europe, Zwijndrecht, Belgium) and were used without further purification. [Cp*CoI_2_]_2_ was synthesized as reported in the literature [55,56].

NMR spectra were measured on a Bruker DRX 500, Bruker AMX 400, Bruker DRX 300 (Billerica, MA, USA) or Varian Mercury 300 spectrometer (Palo Alto, CA, USA) at 298 K unless otherwise stated, and the reported ppm values are relative to SiMe_4_, by referencing the solvent residual peak to SiMe_4_. Individual peaks are reported as: multiplicity (s = singlet, d = doublet, t = triplet, q = quartet, and m = multiplet), integration, and coupling constant (J) in Hz. Data were processed and visualized using MestReNova 15.0.1.

Mass spectra were collected on a HR–ToF Bruker Daltonik GmbH (Bremen, Germany) Impact II, an ESIToF MS capable of a resolution of at least 40,000 FWHM, which was coupled to a Bruker cryo-spray unit. The source voltage was between 3 and 6 kV. The sample was introduced with a syringe pump at a flow rate of 180 μL/h. The drying gas (N_2_) was held at −40 °C, and the spray gas was held at −35 °C. Software acquisition: Compass 2.0 for Otof series. Spectra were visualized using mMass 5.5.0.

UV/Vis spectra were recorded on a single-beam Hewlett Packard 8453 spectrometer (Agilent Technologies, Inc., Santa Clara, CA, USA) in a 1.0 cm quartz cuvette using the solvent as background at 20.0 °C, unless otherwise stated.

Infrared (IR) spectra were measured on a Bruker Alpha-P FT–IR instrument in the ATR geometry with a diamond ATR unit.

Single-crystal X-ray diffraction data of [Cp*Co(4DHBP)I] were measured on a Bruker D8 Quest Eco diffractometer using graphite-monochromated (Triumph) Mo Ka radiation (λ = 0.71073 Å) and a CPAD Photon III C14 detector (Bruker, Billerica, MA, USA). The sample was cooled with N_2_ to 100 K with a Cryostream 700 (Oxford Cryosystems, Oxfordshire, UK). Intensity data were integrated using the SAINT V8.42 software [57]. Absorption correction and scaling were executed with SADABS 2016/2 [58]. The structures were solved using intrinsic phasing with the program SHELXT 2018/2 [59] against *F*^2^ of all reflections. Least-squares refinement was performed with SHELXL-2019/2 [60]. All non-hydrogen atoms were refined with anisotropic displacement parameters. The hydroxyl hydrogens were placed using HFIX 88 and a DFIX command. The hydrogen atoms were introduced at calculated positions with a riding model. The crystal structure contained four voids (total solvent accessible volume = 995 Å^3^), containing CH_2_Cl_2_ solvent within the asymmetric unit, which could not be refined reliably. Thus, the SQUEEZE [61] procedure in PLATON [62] (version 260325) was applied, accounting for 662 electrons per unit cell, congruent with the presence of 1 CH_2_Cl_2_ molecule (42 e-/molecule) in the unit cell (*Z* = 16). CheckCIF revealed no A-level alerts. The X-ray crystallographic data for [Cp*Co(4DHBP)I] (2546192) were deposited at the Cambridge Crystallographic Data Centre (CCDC).

HCOO^−^ detection and quantification were carried out by ^1^H NMR analysis of 0.2 mL of the reaction mixtures by addition of 0.3 mL of D_2_O for locking purposes using 1-butanol as internal standard. MeOH detection and quantification were carried out on a Shimadzu 2010 Pro gas chromatograph (GC) (Nakagyo-ku, Japan) equipped with a fused silica porous polymer PLOT column (30 m × 0.32 mm i.d., film thickness 10 μm, SH-Rt-U-BOND). A flame ionization detector (FID) was applied for the detection of target molecules. The experiments were conducted under the following conditions: an inlet temperature of 250 °C, a column pressure of 128.7 kPa, a total flow rate of 66.0 mL·min^−1^, a detector temperature of 200 °C, and a split ratio of 20. The column heating program was as follows: 150 °C for 10 min, ramp to 170 °C (5 °C min^−1^), 170 °C for 2 min, ramp to 180 °C (5 °C min^−1^), 180 °C for 5 min, ramp to 185 °C (2 °C min^−1^), and 185 °C for 20 min. Before and after each run, a clean sample containing a 4:1 mixture of H_2_O/THF was injected to ensure no cross-contamination between runs.

### 3.2. Synthetic Procedures

#### 3.2.1. Synthesis of [Cp*CoI(4,4′-diol-2,2′bipyridyl)]I

To a 50 mL Schlenk flask equipped with stirring bar, [Cp*CoI_2_]_2_ (100 mg, 1 eq, 112 μmol) and [2,2′-bipyridine]-4,4′-diol (42.0 mg, 2 eq, 223 μmol) were added, and the flask was cycled with vacuum-argon three times. Under Ar via syringe, dry DCM (10 mL) was added. The dark-green solution was stirred at room temperature for 18 h.


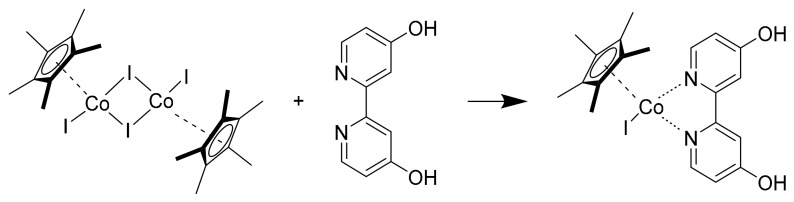


After the reaction, dry pentane (20 mL) was added via syringe under Ar, and the reaction mixture was stored at −20 °C for 18 h. After this time, the solution was removed from the solid via syringe under Ar, and the obtained dark red solid was washed with pentane (10 mL × 2) and dried under high vacuum (72.8% yield). The compound was paramagnetic, as deduced from attempts at recording a ^1^H NMR spectrum. The product was characterized by ESI–MS in its doubly deprotonated form (calculated [M + 1] = 381.1013; measured mass [M + 1] = 381.1014). Crystals suitable for single-crystal X-ray diffraction were obtained by layering a saturated DCM solution with hexane.

Crystal data for C_21_H_25_Cl_2_CoIN_2_O_2_ (M = 594.16 g/mol): orthorhombic, space group Pnna (no. 52), a = 14.0589(4) Å, b = 35.3601(9) Å, c = 16.9765(5) Å, a = β = g = 90°, V = 8439.4(4) Å^3^, *Z* = 16, T = 100(2) K, *μ*(MoKα) = 2.551 mm^−1^, *ρ*_calc_= 1.871 g cm^−3^, and 179,165 reflections measured (8.17° ≤ 2Θ ≤ 50.05°), 7427 of which were unique (*R*_int_ = 0.1012, *R*_sigma_ = 0.0259), which were used in all calculations. The final R_1_ was 0.0345 (I ≥ 2σ(I)), and wR_2_ was 0.0938 (all data).

ATR–IR (cm^−1^): 1600 ν(C-C,C-N); 1519 ν(C-C,C-N); 1427 ν(C-C,C-N); 1366 δ(C-H); 1263, 1234 ν(C-CH_3_, C-H); 1038 δ_in-plane_(C-H, ring); 1015 ρ(CH_3_); 990 γ(C-H); 918 ν(C-CH_3_, C=O); 877 γ(C-H); 855 γ(C-H); 817, 745 δ(COO, C-H); 694 φ(CC), φ(CN); 578, 480, 433 δ(C-CH_3_).

ε (20 °C, 239 nm, Britton–Robinson buffer 20 mM) = 5542.8 M^−1^ ∙ cm^−1^.

ε (20 °C, 525 nm, Britton–Robinson buffer 20 mM) = 76.37 M^−1^ ∙ cm^−1^.

#### 3.2.2. UV/Vis Titration of [Cp*CoI(4,4′-diol-2,2′bipyridyl)]I

First, 20 mL of 20 mM Britton–Robinson buffer was prepared by mixing 20 mL of MilliQ water with phosphoric acid (66 mg, 39 μL, 0.90 Eq, 0.67 mmol), acetic acid (44 mg, 42 μL, 0.98 Eq, 0.73 mmol) and boric acid (46 mg, 33 μL, 1 Eq, 0.74 mmol) [63,64].

Next, 2 mL of the buffer was added to a quartz UV/Vis cuvette with 1 cm path length for the reference sample. Then, 2 mL of the buffer was added to a second quartz cuvette, to which 10 µL of an aqueous solution of [Cp*CoI(4,4′-diol-2,2′bipyridyl)]I (0.38 g, 1 Eq, 0.74 mmol) was added. The measured species is therefore [Cp*Co(H_2_O)(4,4′-diol-2,2′bipyridyl)].

Both mixtures were titrated with sequential additions of 0.5 M NaOH in MilliQ water so that the pH ranged from 2 to 10 (addition of 78 µL to increase the pH of 1 unit). After every addition to both cuvettes, UV/Vis spectra were recorded at 20 °C (Figure S2).

By tracking the change in absorbance at different pH levels, two deprotonation steps were identified at approximately pH 4.5 and 9 (Figure S2), attributed, respectively, to the deprotonation of the hydroxyl groups and to the deprotonation of the aqua ligand [45]. The first deprotonation step was calculated to have a pk_a_ of 4.14 (Figure S3).

#### 3.2.3. Synthesis of [Cp*CoI(5,5′-dimethyl-2,2′bipyridyl)]I

To a flame-dried 25 mL Schlenk flask equipped with stirring bar and a J. Young valve, [(Cp*CoI_2_)_2_] (100 mg, 0.5 Eq, 112 μmol) and 5,5′-dimethyl-2,2′-bipyridine (47 mg, 1.1 Eq, 0.26 mmol) were added under Ar. DCM (20 mL) was added to the flask via syringe under Ar, and the dark-brown reaction mixture was stirred at 35 °C for 18 h.


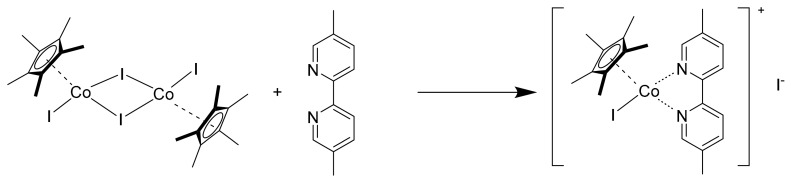


After the reaction, the mixture appeared as a dark-brown solution. The mixture was filtered on a syringe filter under Ar and was layered with pentane (20 mL) and kept at 4 °C for 10 days to obtain a dark solid. The solid was removed from the solution and dried in high vacuum. [Cp*CoI(5,5′-dimethyl-2,2′-bipyridyl)]I (140 mg, 221 μmol, 99.2%) was obtained as a dark-purple solid.

^1^H NMR (400 MHz, Chloroform-*d*) δ 9.10–8.95 (m, 1H), 8.59 (d, *J* = 8.3 Hz, 1H), 8.34–7.91 (m, 1H), 2.59 (s, 3H), 1.60 (s, 8H).

#### 3.2.4. Synthesis of [Ni(4DHBP)(acac)] and [Ni(4DHBP)_2_]

Ni(acac)_2_ (20 mg, 1 Eq, 78 μmol), [2,2′-Bipyridine]-4,4′-diol (15 mg, 1 Eq, 78 μmol) and sodium bicarbonate (6.5 mg, 1 Eq, 78 μmol) were added to a 25 mL Schlenk flask equipped with a stirring bar. The flask was cycled three times with N_2_/vacuum. Under N_2_, water (4 mL) and THF (1 mL) were added via syringe. The reaction mixture was stirred at 80 °C for 18 h under N_2_.


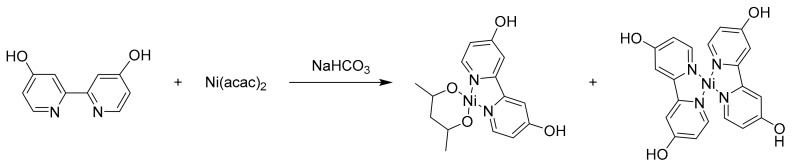


After the reaction, the mixture appeared as a pale-pink suspension, with an off-white solid. The liquid was removed via syringe and filtered on a hydrophilic syringe filter into a separate Schlenk flask under N_2_. The filtrate was dried under high vacuum to obtain an off-white powder, which was analyzed by ^1^H NMR and ESI–MS.

From ^1^H NMR analysis, it can be deduced that some coordination of the bipyridine ligand to the nickel has occurred. The broad peaks are due to the paramagnetic nature of the Ni^II^ species.

From ESI–MS analysis (in H_2_O), it can be deduced that both a mono-bipyridine complex and a bi-bipyridine complex had been formed in the reaction mixture (respectively, calculated [M] = 345.039 and measured mass [M] = 345.0383, and calculated [M] = 434.052 and measured mass [M] = 434.0492) along with the singly deprotonated [Ni(4DHBP)_2_] species (calculated [M]= 433.045; measured mass [M]= 433.0441) and the sodium salt of the deprotonated species (calculated [M] = 455.0261; measured mass [M] = 455.0261).

ATR–IR (Ni(acac)_2_, cm^−1^): 3403 ν(OH, H_2_O); 3076 ν(C-H, CH); 2990 ν(C-H, CH_3_); 2925 ν(C-H); 1654 ν(CC,CN); 1590 ν_as_(OCO); 1510 ν_as_(CC,CN); 1462 δ(C-H); 1394 δ(C-H); 1261 ν(C-CH_3_, C-H); 1201 δ(C-H); 1018 ρ(CH_3_); 933 ν(C-CH_3_, C=O); 765 δ(COO, C-H); 675, 662 ν(M-L); 588 ν(M-O); 575 δ(C-CH_3_).

ATR–IR (Ni(4DHBP)_2_ crude mixture, cm^−1^): 2961 ν(C-H, CH_3_); 2922 ν(C-H); 2853 ν(C-H)_OOCH_; 1591 ν_as_(OCO); 1420 δ(C-H); 1259 ν(C-CH_3_, C-H); 1080 δ_in-plane_(C-H, ring); 1013 ρ(CH_3_); 878 γ(C-H); 797 δ(COO, C-H); 694 φ(CC), φ(CN); 626 ν(M-L).

UV/Vis (Ni(acac)_2_): λ_max_= 295.5 nm.

UV/Vis (4DHBP): λ_max_= 232.5 nm.

UV/Vis (Ni(4DHBP)_2_): λ_max_= 243 nm.

The reaction mixture was then used to test the possibility of the formation of Ni-H.

#### 3.2.5. Hydrogenation of [Ni(4DHBP)_2_]

The reaction mixture of the previous experiment was connected through a T-piece to a Schlenk line and a closed H_2_ 3 L balloon at 1 bar. The mixture was cycled through a freeze–pump–thaw cycle 3 times under static vacuum (Schlenk line tap closed), and then the balloon was opened to obtain a pure H_2_ atmosphere. The reaction was stirred under H_2_ at 25 °C for 18 h. The temperature was increased to 50 °C for 2 h and then 60 °C for 18 h.

After the reaction, the mixture appeared as a pale-pink solution, and the balloon of hydrogen appeared significantly deflated. The solvent was evaporated under high vacuum using an external cold trap.

From ^1^H NMR analysis in CD_3_OD, no hydride was observed in the region 0 to −40 ppm, likely due to paramagnetism of the species formed. ESI–MS analysis revealed a difference in the ratio of the species corresponding to 434 and 433 *m*/*z*. This could be due to protonation of the 4DHBP backbone or to hydride formation. From ATR–IR analysis, no hydride formation was observed. Formation of Ni-H in these conditions was therefore ruled out.

#### 3.2.6. Synthesis of [Ni(4HDBP)(OOCH)]

In air, sodium formate (31 mg, 10 Eq, 0.46 mmol) was added to a Schlenk flask and was cycled with vacuum and argon three times before addition of water (3 mL). The sodium formate solution was added under Ar via syringe to the Schlenk flask containing the previously described [Ni(4DHBP)_2_] mixture, and the cloudy-white suspension was left under stirring at 25 °C for 18 h.


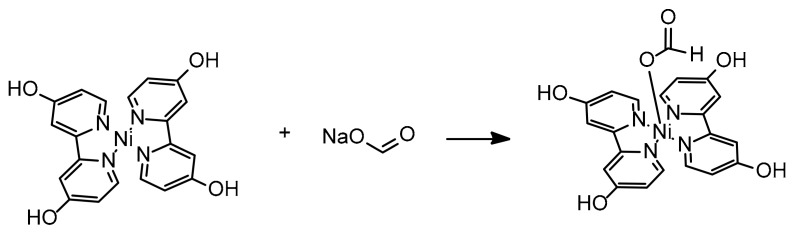


After the reaction, the solvent was evaporated in vacuo using an external cooling trap. The obtained light-pink solid was then washed with DCM (10 mL) and then dried under high vacuum.

From ESI–MS analysis, the corresponding peak for [Ni(4DHBP)_2_] was detected (calculated [M] = 433.045; measured mass [M] = 433.0460). A smaller peak corresponding to [Ni(4DHBP)_2_(OOCH)] was also detected and identified (calculated [M] = 479.0502; measured mass [M] = 479.0501). A smaller peak corresponding to [Ni(4DHBP)_3_] was also identified (621 *m*/*z*).

ATR–IR (Ni(4DHBP)_2_(OOCH), cm^−1^): 2977 ν(C-H, CH_3_); 2832 ν(C-H)_OOCH_; 1615 ν_as_(OCO); 1427 δ(C-H); 1372 δ(C-H); 1341 ν_s_(OCO); 1261 ν(C-CH_3_, C-H); 1077 δ_in-plane_(C-H, ring); 1011 ρ(CH_3_); 878, 826 γ(C-H); 774, 766 δ(COO, C-H); 671 φ(CC), φ(CN); 665 ν(M-L); 584 ν(M-O).

UV/Vis: λ_max_= 242.5 nm.

### 3.3. General Catalysis Procedure

#### 3.3.1. Catalytic Hydrogenation of CO_2_

The reaction reagents were added inside a glovebox (N_2_ atmosphere) to a glass insert equipped with stirring bar, and the insert was capped. An autoclave containing 4 stainless steel reactors of 25mL capacity separated by taps was flushed with Ar for 30 min. Under a N_2_ flow, the reaction mixtures were loaded in each reactor, the solvent mixture was added via syringe, and the tap between the reactor and the common line was closed. The vessel was pressurized with 10 bar of carbon dioxide and subsequently 40 bar of dihydrogen (unless otherwise stated), to reach a total pressure of 50 bar. All reactors were pressurized by opening their respective taps one by one. The closed vessel was heated to 100 °C for 24 h while stirring (unless otherwise stated). After the reaction, the autoclave was transferred to an ice-water bath for 30 min, and then each reactor was very carefully depressurized while flushing the common line in between each depressurization with N_2_ to prevent any cross-contamination. Butan-1-ol (10 μL, 109 μmol) was added as an internal standard, and 0.3 mL of the reaction mixture was added to an NMR tube alongside 0.2 mL of D_2_O for locking purposes. HCOO^−^ was quantified by ^1^H NMR (300 MHz, 25 °C, D_2_O). GC samples were prepared by filtering the reaction mixture through a plug of celite after addition of the internal standard. Calibration of the GC integration for quantification of methanol was also performed with filtered samples. Methanol was quantified by GC analysis. The formation of formate esters was never detected by GC analysis.

#### 3.3.2. Ligand Scrambling in Catalytic Conditions

The general catalysis procedure for hydrogenation of CO_2_ was followed using [Cp*Co(4DHBP)I] 3 µmol, [Ni(acac)_2_] 3 µmol, NaHCO_3_ 4 mmol, pCO_2_ 10 bar, pH_2_ 40 bar, and 5 mL H_2_O/THF (4:1) at 100 °C for 24 h. After the reaction, the crude reaction mixture was analyzed by ESI–MS. A species corresponding to [Ni(acac)(4DHBP)-H]^+^ was detected (calculated [M] = 345.039; measured mass [M] = 345.0383), confirming ligand scrambling during the catalytic reaction.

## 4. Conclusions

In this work, a cooperative, dinuclear mode of activation for the formate intermediate in the hydrogenation of CO_2_ to methanol is presented. A new [Cp*Co(4DHBP)I] precatalyst was synthesized and characterized. Combined with [Ni(acac)_2_], production of both formate and methanol achieved up to 13.2 and 12.1 TON, respectively. Using a nickel complex instead of the previously reported additives (acids, amines, and alcohols) presents the advantage that small amounts of additive are required for catalytic production of methanol. The possibility of scrambling of the 4DHBP ligand was investigated and confirmed, although its influence on methanol production was proven superfluous. By probing the system with Ni(OOCH)_2_·2H_2_O, the proposed cooperative dinuclear hydride transfer between a cobalt-hydride species and a nickel–formato species was corroborated, suggesting that the free formate intermediate is activated by coordination with the nickel center. Further studies are needed to explore the detailed mechanism of this activation and whether it is correlated with the free energy of the nickel–formato species. By studying this activation mode in more detail, further improvements to this system could be better explored.

## Figures and Tables

**Table 1:** Hydrogenation of CO_2_ to HCOO^−^ and CH_3_OH by [Cp*Co(4DHBP)I] and [Ni(acac)_2_] combined ^1^.

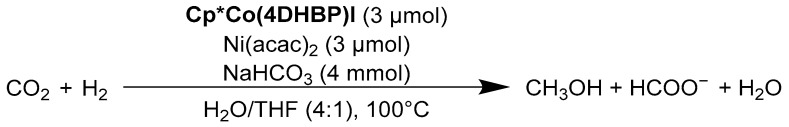			
Entry	Variation	HCOO^−^ TON	CH_3_OH TON
1	No Ni(acac)	92	1.6
2	No [Cp*Co(4DHBP)I]	1.6	0.3
3	No CO_2_	-	-
4	None	4.2	4.0
5	[Cp*Co(4DHBP)I] 10 µmol	1.0	1.7
6	Ni(acac)_2_ 12 µmol	13.2	12.1
7	Ni(COD)_2_ 10 µmol	5.9	2.9
8	NaHCO_3_ 2 mmol	1.1	3.5

^1^ Standard conditions: [Cp*Co(4DHBP)I] 3 µmol, [Ni(acac)_2_] 3 µmol, NaHCO_3_ 4 mmol, pCO_2_ 10 bar, pH_2_ 40 bar, 5 mL H_2_O/THF (4:1), 100 °C, 24 h. Estimated pH in standard conditions: 8.6. HCOO^−^ TON was calculated by ^1^H NMR using 1-butanol as internal standard, referenced to [Cp*Co(4DHBP)I]. CH_3_OH TON was calculated by GC using 1-butanol as internal standard, referenced to [Cp*Co(4DHBP)I]. No other species were detected.

## Data Availability

The raw data supporting the conclusions of this article will be made available by the authors on request.

## Supplementary Materials

The following supporting information can be downloaded at: https://www.mdpi.com/article/10.3390/molecules31122047/s1. SI. Description of the reactors. SII. Additional catalysis experiments. SIII. ATR–IR data. SIV. Recorded spectra. SV. Crystallographic data. CCDC 2546192 contains the supplementary crystallographic data for this paper. These data can be obtained free of charge via https://www.ccdc.cam.ac.uk/structures/ (or from the CCDC, 12 Union Road, Cambridge CB2 1EZ, UK; Fax: +44 1223 336033; E-mail: deposit@ccdc.cam.ac.uk).

## Abbreviations

The following abbreviations are used in this manuscript:

2MeTHF	2-methyltetrahydrofuran
4DHBP	4,4′-dihydroxy-2,2′-bipyridine
acac	Acetylacetone
ATR	Attenuated total reflectance
bipy	2,2′-bipyridine
Cp*	1,2,3,4,5-pentamethylcyclopentadienyl
Cp*Co(4DHBP)I	[(1,2,3,4,5-pentamethylcyclopentadienyl)cobalt(II)iodo(4,4’-diol-2,2’bipyridyl)]iodide
Cp*Co(5Me_2_bipy)I	[Cp*CoI(5,5’-dimethyl-2,2’bipyridyl)]I
DCM	Dichloromethane
ESI–MS	Electrospray ionization–mass spectrometer
EtOH	Ethanol
FID	Flame ionization detector
FT–IR	Fourier transform–infrared
GC	Gas chromatography
HCOOEt	Ethyl formate ester
HCOONa	Sodium formate
MeOH	Methanol
Ni(COD)_2_	Bis(cyclooctadiene)nickel(0)
NMR	Nuclear magnetic resonance
SiMe_4_	Trimethylsilane
THF	Tetrahydrofuran
TON	Turnover number
Triphos	1,1,1-Tris(diphenylphosphinomethyl)ethane
UV/Vis	Ultraviolet–visible spectroscopy
γ	Bend out-of-plane
δ	Scissoring
ν	Stretching
ρ	Rocking in-plane
φ	Torsion
ΔG°	Gibbs free energy of formation
