# Supplementary material for: Cooperative Dinuclear Activation of a Formate Intermediate in the Hydrogenation of CO2 to Methanol
Source: Molecules. 2026 Jun 11;31(12):2047. doi: 10.3390/molecules31122047 (PMC13304865; doi:10.3390/molecules31122047)
Supplement: Supplementary file 1 [file molecules-31-02047-s001.zip › molecules-4319457-supplementary.pdf]

Contents

SI. Description of the reactors 2

SII. Additional catalysis experiments 3

SII.A Catalytic activity of [Cp*Co(4DHBP)I] 3

SII.B Catalytic activity of [Ni(acac)$_2$] 3

SII.C Catalytic activity time dependence 4

SII.D Catalytic activity of [Cp*CoI(5,5'-dimethyl-2,2'bipyridyl)]I 4

SII.E Catalytic hydrogenation of formaldehyde 4

SIII. ATR–IR data 6

SIV. Recorded spectra 7

SV. Crystallographic data 16

SI. Description of the reactors

For high-pressure catalysis experiments, cylindrical stainless steel vessels produced in-house were utilized. Internal diameter 20 mm, internal height 80 mm and total internal volume 25 mL. 4 of these reactors were connected to a main line and separated from it by one tap for each vessel and were pressurized one by one. To ensure no cross-contamination between vessels, the taps remained closed during the reaction. In addition to that, extensive flushing with N_2 with the taps closed was performed in the common line between the depressurization of each vessel. A manometer and a safety pressure-relief valve were installed on the common line. The reactors and the common line was leak-tested each time pressure was applied to the autoclave.

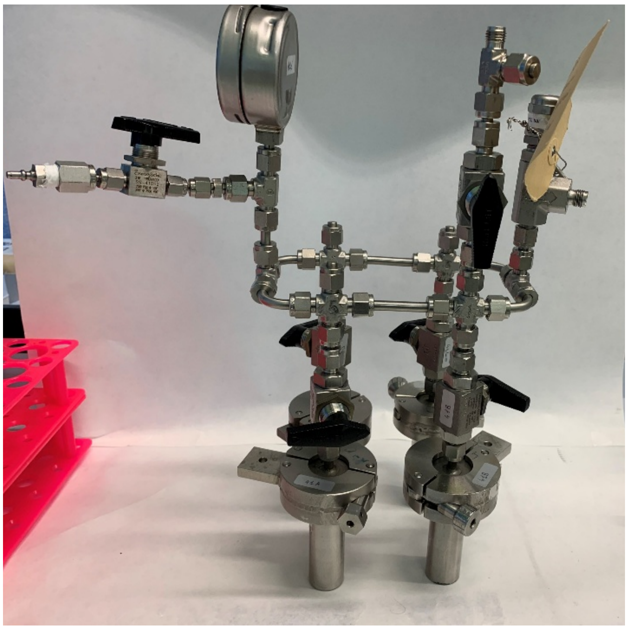

Figure S1: High-pressure autoclave consisting of 4 25 mL reaction vessels connected to a main line by taps and a manometer and safety pressure-relief valve.

Cylindrical stirring bars of 3 mm length were used for magnetic stirring, with 800 revolutions per minute as the stirring speed. The reactors were in a single silicone oil bath on a single hotplate. The time for the inner part of the reactor to reach the set heating temperature was estimated to be 30 minutes, therefore the start of the reaction time was set 30 minutes after the heating was first applied. The reactors were cooled in an ice-water bath for 30 minutes before depressurizing.

The following cleaning procedure was performed after each autoclave run:

After initial washing with THF and water, the inside of the autoclave pot was cleaned mechanically with brush and abrasive detergent, rinsed with deionized water and air-dried. The autoclave head was rinsed extensively with THF through all accessible openings and blown dry with pressurized air.

To ensure no metal contamination on the surface of the stainless steel, the following cleaning procedure was performed after autoclave runs consisting of metals differing from Ni and Co:

2.0 g triphenylphoshine and 50 mL toluene were filled into the autoclave pot equipped with a magnetic stirring bar. After flushing the autoclave with H_2 three times up to 5 bar, it was filled with H_2 to 15 bar and heated at 120°C for 18 h while stirring with each of the taps to the vessels open. After cooling, the standard cleaning procedure was performed.

The validity of the cleaning procedure was checked by a blank run containing only the solvent.

SII. Additional catalysis experiments

SII.A Catalytic activity of [Cp*Co(4DHBP)I]

Table S1: Hydrogenation of CO_2 to $HCOO^-$ and MeOH by ***[Cp*Co(4DHBP)I]****.*

$$CO_2 + H_2 \xrightarrow[H_2O/THF\ (4:1),\ 100°C]{\textbf{Cp*Co(4DHBP)I}\ (3\ \mu mol),\ NaHCO_3\ (4\ mmol)} HCOO^- + CH_3OH$$

Entry	Deviation	TON $HCOO^-$	TON CH_3OH
**1**	none	92	1.56
**2**	80°C	4.5	0.00
**3**	only H_2O	70	0.00
**4**	in EtOH	traces	1.97
**5**	+70 µmol MeOH	11	-
**6**	8 mmol $NaHCO_3$	14	0.00
**7**	2 mmol $NaHCO_3$	10	0.00
**8**	no CO_2	0.00	0.00
**9**	**Cp*Co(5Me_2bipy)I**	0.00	0.00
**10**	THF/H_2O alone	0.00	0.00
**11**	THF/H_2O + $NaHCO_3$ alone	0.00	0.00

Standard conditions: **Cp*Co(4DHBP)I** 3 µmol, $NaHCO_3$ 4 mmol, pCO_2 10 bar, pH_2 40 bar, 5 mL H_2O/THF (4:1), 100°C, 24h. Estimated pH in standard conditions: 8.6. TON $HCOO^-$ calculated by ^1H NMR using 1-butanol as internal standard. MeOH TON calculated by GC using 1-butanol as internal standard. No other species were detected.

SII.B Catalytic activity of [Ni(acac)$_2$]

Table S2: Hydrogenation of CO_2 to $HCOO^-$ and MeOH by [Ni(acac)$_2$].

$$CO_2 + H_2 \xrightarrow[H_2O/THF\ (4:1),\ 100°C]{Ni(acac)_2\ (0.03\ mmol),\ NaHCO_3\ (4\ mmol)} CH_3OH + HCOO^- + H_2O$$

Entry	Deviation	TON $HCOO^-$	TON CH_3OH
**1**	none	1.57	0.31
**2**	no CO_2	0.00	0.00
**3**	+ 0.01 mmol 4DHBP	3.76	1.02
**4**	Ni(COD)$_2$	0.50	1.00
**5**	Ni(COD)$_2$ + 0.05 mmol acac	0.17	0.69
**6**	Ni(COD)$_2$ + 0.01 mmol 4DHBP	0.30	1.21

Standard conditions: Ni(acac)$_2$ 30 µmol, $NaHCO_3$ 4 mmol, pCO_2 10 bar, pH_2 40 bar, 5 mL H_2O/THF (4:1), 100°C, 24h. Estimated pH in standard conditions: 8.6. TON $HCOO^-$ calculated by ^1H NMR using 1-butanol as internal standard. MeOH TON calculated by GC using 1-butanol as internal standard. No other species were detected.

SII.C Catalytic activity time dependence

*Table S3: Hydrogenation of CO_2 to $HCOO^-$ and CH_3OH by **[Cp*Co(4DHBP)I]** and [Ni(acac)$_2$] combined.*

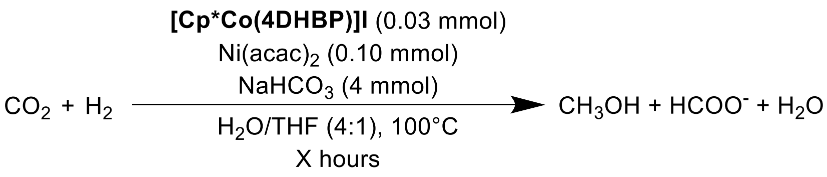

Entry	Time	TON HCOO$^-$	TON CH_3OH
**1**	3 hours	3.5	5.1
**2**	6 hours	0	4.6
**3**	18 hours	12.2	6.9
**4**	24 hours	13.2	12.1

Standard conditions: **[Cp*Co(4DHBP)I]** 3 µmol, [Ni(acac)$_2$] 10 µmol, $NaHCO_3$ 4 mmol, pCO_2 10 bar, pH_2 40 bar, 5 mL H_2O/THF (4:1), 100°C, variable reaction time. Estimated pH in standard conditions: 8.6. $HCOO^-$ TON calculated by ^{1}H NMR using 1-butanol as internal standard, referenced to **[Cp*Co(4DHBP)I]**. CH_3OH TON calculated by GC using 1-butanol as internal standard, referenced to **[Cp*Co(4DHBP)I].** No other species was detected.

SII.D Catalytic activity of [Cp*CoI(5,5'-dimethyl-2,2'bipyridyl)]I

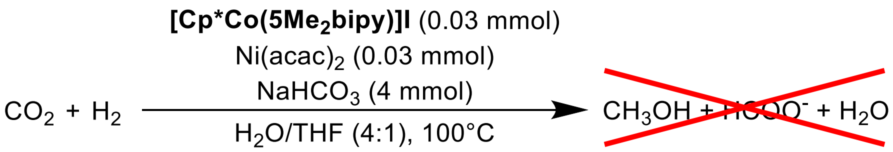

Standard conditions: **[Cp*Co(5Me$_2$bipy)I]** 3 µmol, Ni(acac)$_2$ 3 µmol, $NaHCO_3$ 4 mmol, pCO_2 10 bar, pH_2 40 bar, 5 mL H_2O/THF (4:1), 100°C, 24 h. Estimated pH in standard conditions: 8.6. $HCOO^-$ TON calculated by ^{1}H NMR using 1-butanol as internal standard, referenced to **[Cp*Co(4DHBP)I]**. CH_3OH TON calculated by GC using 1-butanol as internal standard, referenced to **[Cp*Co(4DHBP)I].** No other species was detected.

[Cp*Co(5Me$_2$bipy)I] was tested following the general catalysis procedure for the hydrogenation of CO_2. No products were observed.

SII.E Catalytic hydrogenation of formaldehyde

*Table S4: Hydrogenation of paraformaldehyde catalyzed by **[Cp*Co(4DHBP)]I** and [Ni(acac)$_2$].*

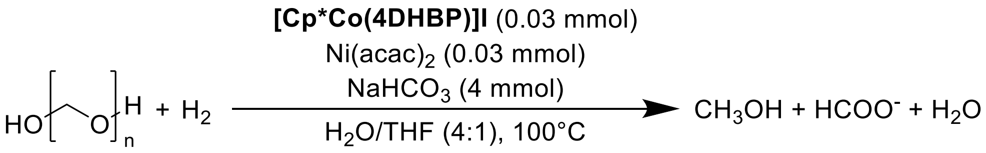

Entry	Deviation	µmol HCOO$^-$	µmol CH_3OH
**1**	none	44	210
**2**	no **[Cp*Co(4DHBP)]I**	42	254
**3**	no Ni(acac)$_2$	42	251
**4**	no **[Cp*Co(4DHBP)]I** no Ni(acac)$_2$	9	79

Standard conditions: paraformaldehyde (0.83 mmol) **[Cp*Co(4DHBP)I]** 3 µmol, [Ni(acac)$_2$] 3 µmol, $NaHCO_3$ 4 mmol, pH_2 40 bar, 5 mL H_2O/THF (4:1), 100°C, 24h. Estimated pH in standard conditions: 8.6. $HCOO^-$ TON calculated by ^{1}H NMR using 1-butanol as internal standard, referenced to **[Cp*Co(4DHBP)I]**. CH_3OH TON calculated by GC using 1-butanol as internal standard, referenced to **[Cp*Co(4DHBP)I].** No other species was detected.

The hydrogenation of formaldehyde to methanol was tested following the general catalysis procedure. Paraformaldehyde was used as a substrate to generate formaldehyde/methandiol in situ in presence of H_2O.

SIII. ATR–IR data

	[Cp*Co(4DHBP)]I	Ni(acac)$_2$	Ni(4DHBP)$_2$	Ni(4DHBP)$_2$(OOCH)	4DHBP	HCOONa	Ni(OOCH)$_2$
**ν (CH)**		3076			3137		
**ν (CH, CH$_3$)**		2990	2961	2977		2941	
**ν (CH)**		2925	2922				2905
**ν (CH) OOCH**			2853	2832		2828	
				2778		2714	
**ν (CO)**							
**ν (CC, CN) bipy**	1600	1654			1614		1671
**$ν_{as}$ (OCO)**		1590	1591	1615	1581	1580	1556
					1558		
**$ν_{as}$ (CC, CN) bipy**	1519	1510			1512		
					1488		
**δ (CH)**		1462		1427	1454		
**ν (CC, CN) bipy**	1427		1420		1435		1398
**δ (CH)**	1366	1394		1372	1361	1354	1372
**$ν_s$ (OCO)**				1341	1336		1352
					1301		
**ν (CCH$_3$, CH)**	1263	1261	1259	1261	1267		
	1234				1228		
**δ (CH)**		1201					
**$δ_{in\text{-}plane}$ (CH)**					1191		
**$δ_{in\text{-}plane}$ (C-H) ring**	1038		1080	1077	1101		
**ρ (CH$_3$)**	1015	1018	1013	1011	1018	1015	
**γ(CH)**	990				993		
**ν (CCH$_3$, C=O)**	918	933			958		
**γ(CH)**	877		878	878	872		889
**γ(CH)**	855				860		852
**γ(CH)**	817			826	826		
**δ (COO, CH)**			797	774	798		795
**δ (COO, CH)**	745	765		766		771	766
**φ(CC), φ(CN)**					739		
	694		694	671	682		
**ν (ML)**		675	626	665	657		
		662		635			
**ν (MO)**		588		584	573		
**δ (CCH$_3$)**	578	575			553		557
	498				529		
	480			477	490		
**ν (MO)**	433				434		

SIV. Recorded spectra

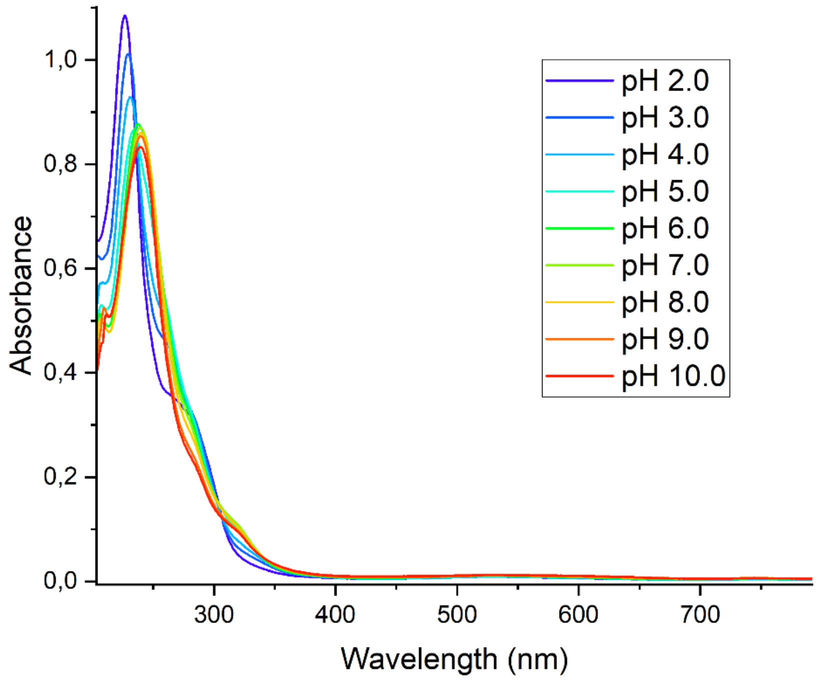

Figure S2: UV/Vis spectrum of ***[Cp*Co(4DHBP)(H_2O)]*** *in 20mM Britton–Robinson buffer at different pH levels.*

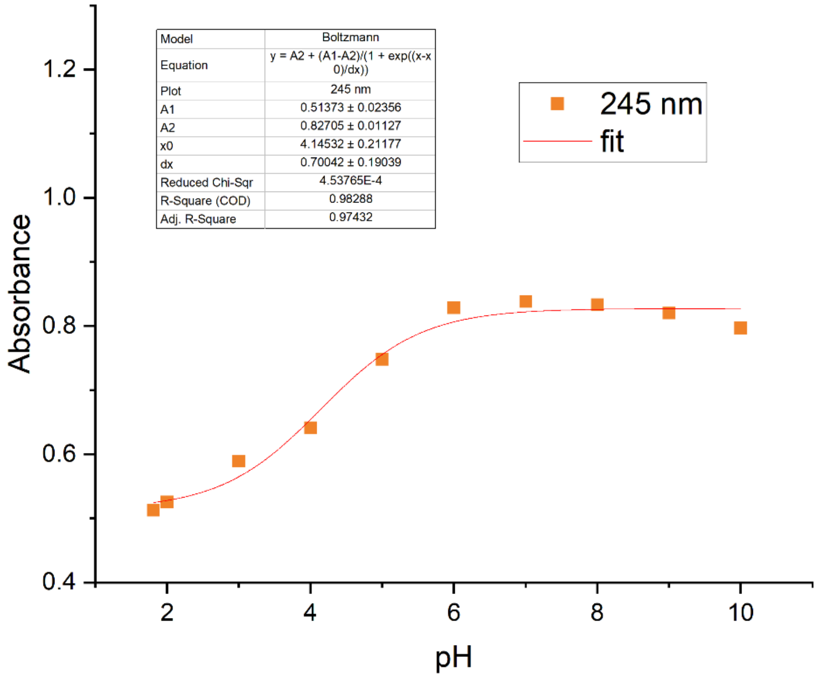

Figure S3: Absorbance change of ***[Cp*Co(4DHBP)(H_2O)]*** *at different pH levels in 20 mM Britton–Robinson buffer 245 nm and fit of the data with a sinusoidal graph. Calculated* pk_a *= 4.14.*

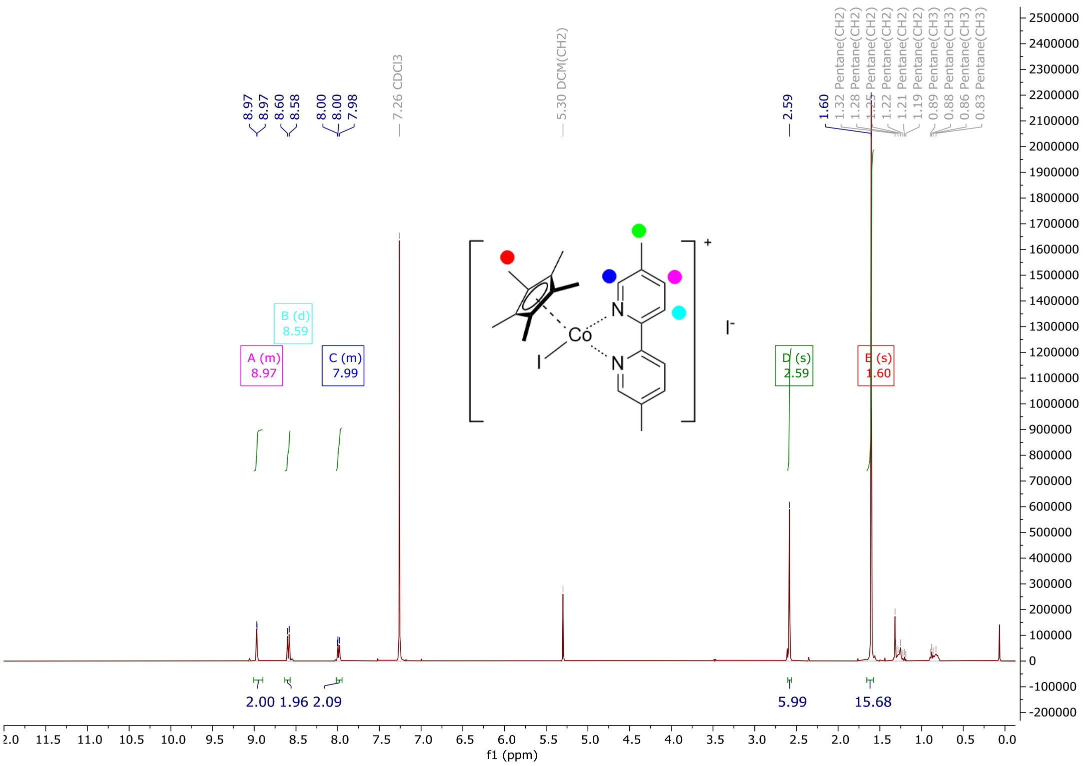

*Figure S4: ^{1}H NMR spectrum of [Cp*CoI(5,5'-dimethyl-2,2'bipyridyl)]I in $CDCl_3$.*

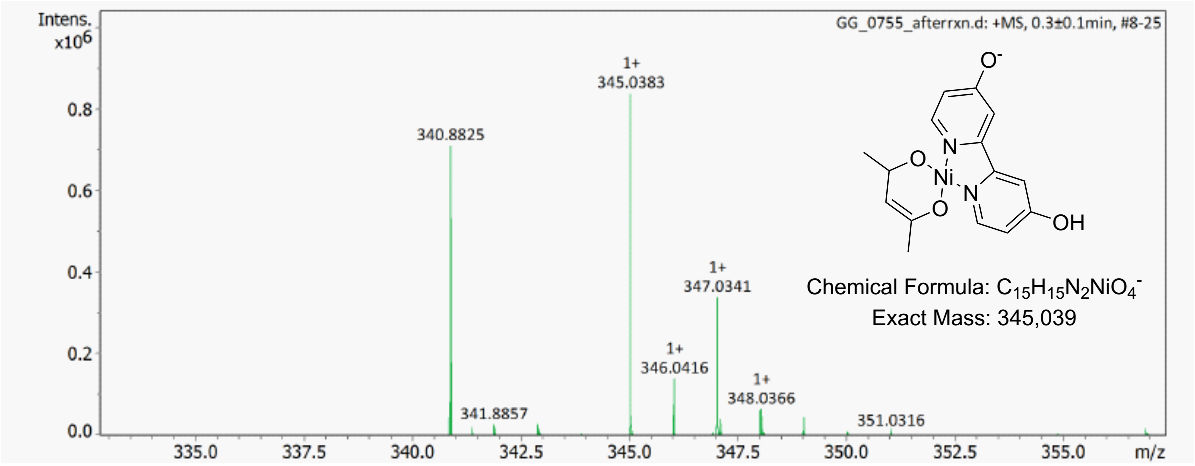

*Figure S5: ESI–MS spectrum of the reaction mixture from a catalytic run containing **[Cp*Co(4DHBP)I]** 3 µmol, [Ni(acac)$_2$] 3 µmol, $NaHCO_3$ 4 mmol, pCO_2 10 bar, pH_2 40 bar, 5 mL H_2O/THF (4:1), 100°C, 24h.*

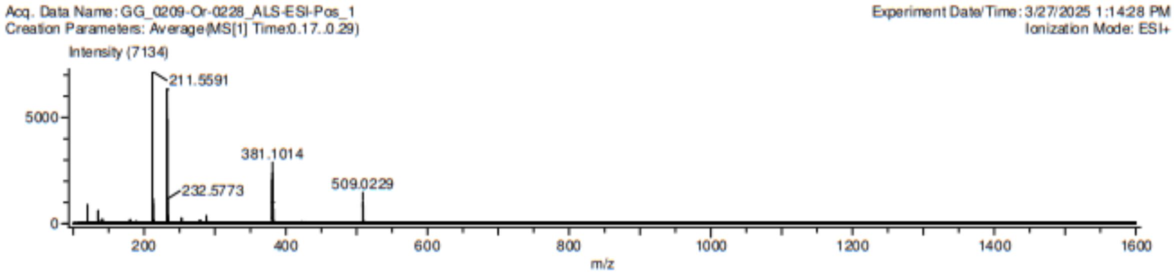

*Figure S6: ESI–MS spectrum of **[Cp*Co(4DHBP)I]**.*

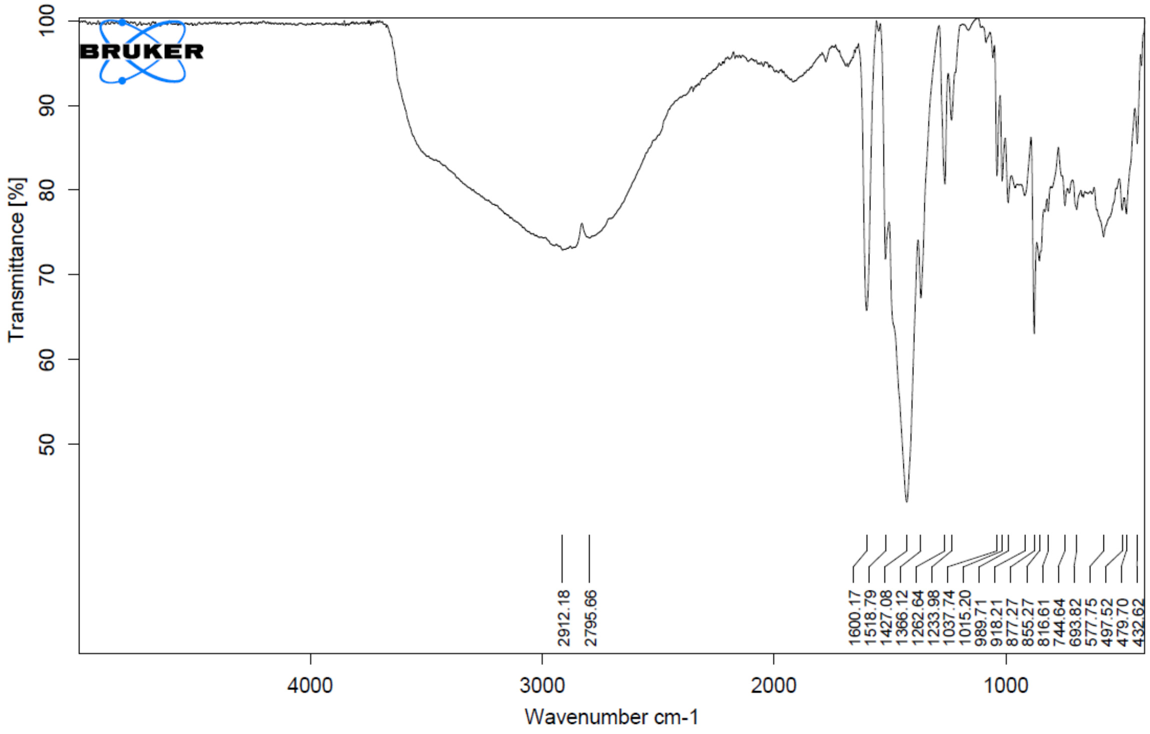

*Figure S7: ATR–IR spectrum of **[Cp*Co(4DHBP)I]**.*

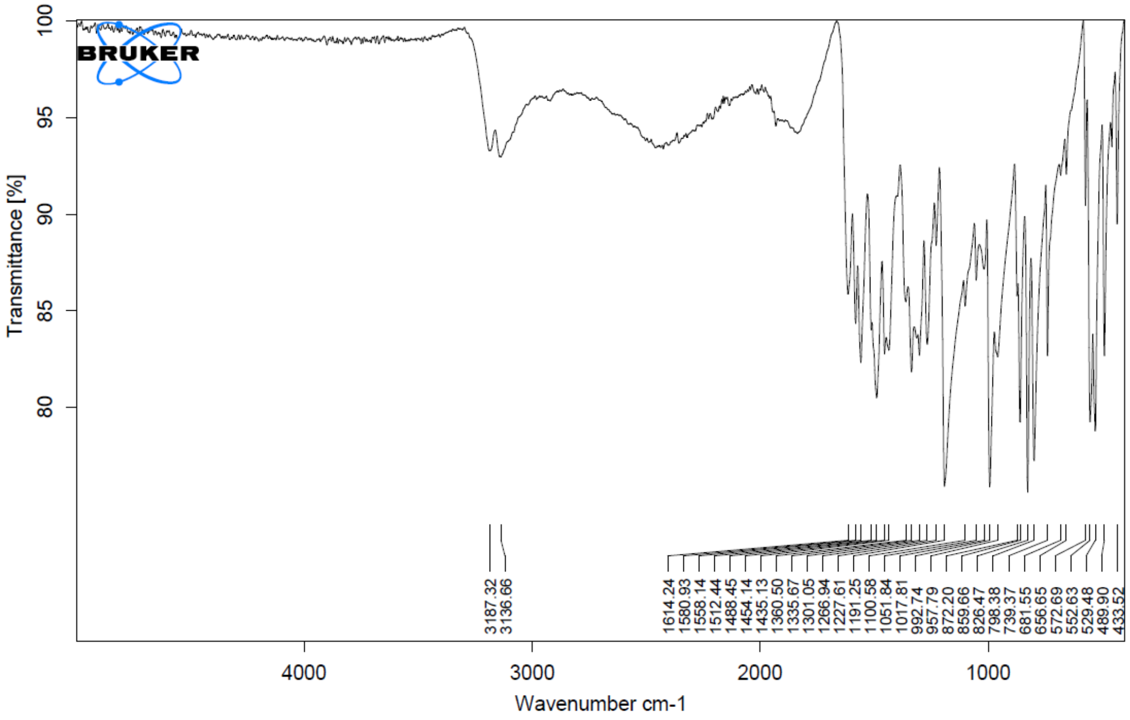

Figure S8: ATR–IR spectrum of 4DHBP.

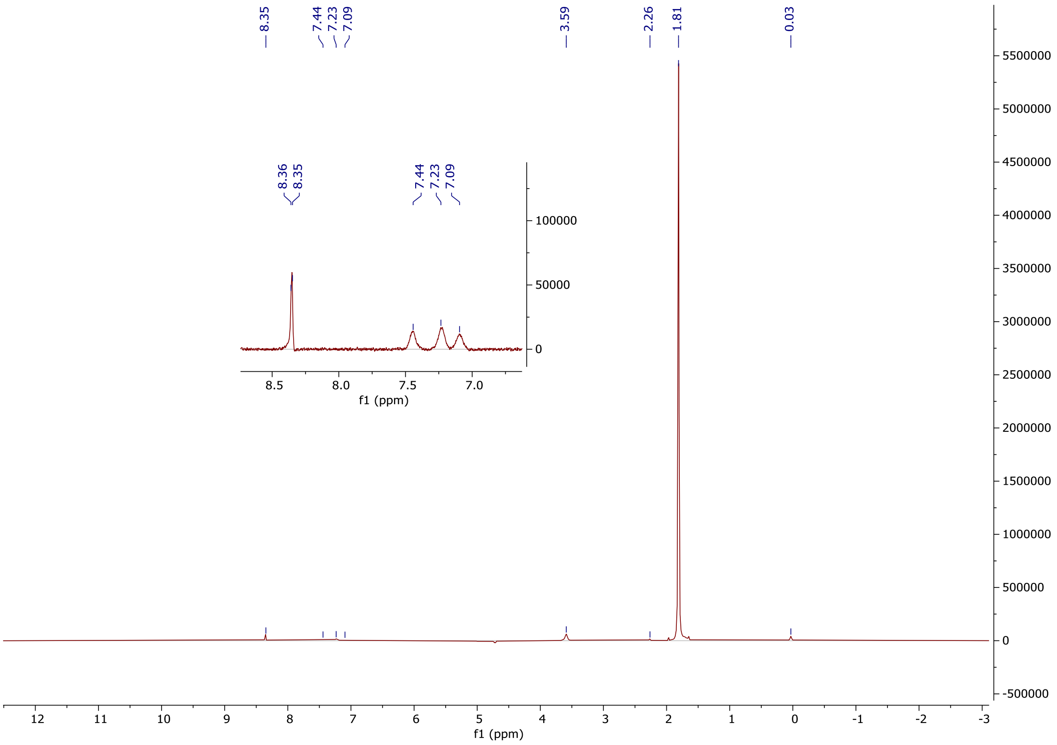

Figure S9: ^{1}H NMR (400 MHz) spectrum of the crude reaction mixture for the synthesis of [Ni(4DHBP)(acac)] and [Ni(4DHBP)$_2$], recorded with solvent suppression (D_2O).

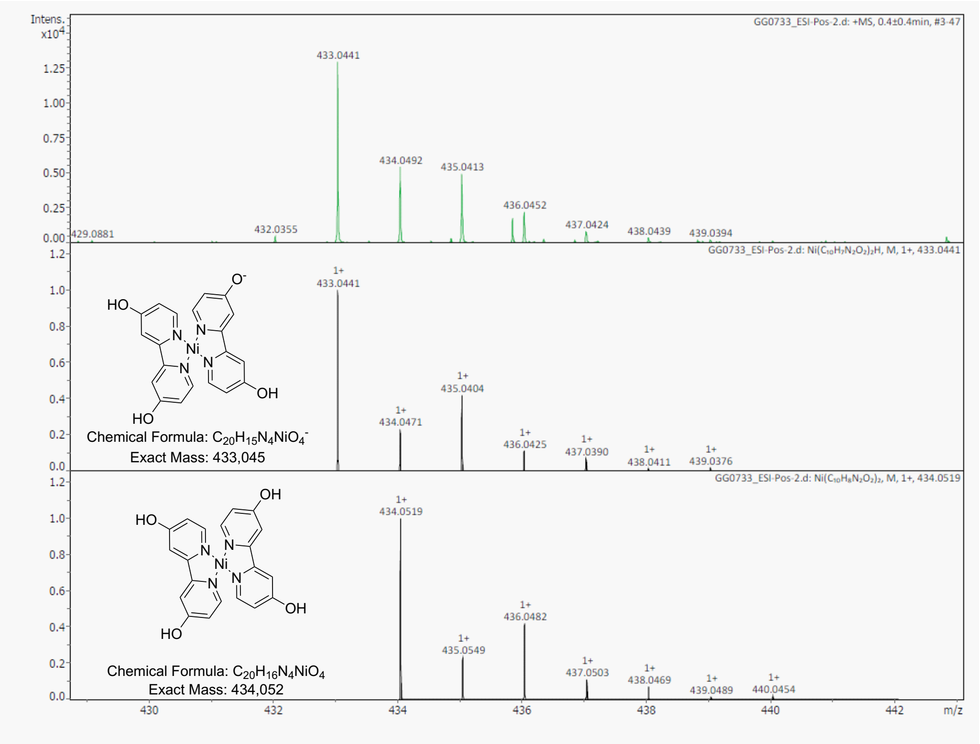

Figure S10: ESI–MS spectrum in H_2O of the reaction mixture for the synthesis of [Ni(4DHBP)(acac)] and $[Ni(4DHBP)_2]$, zoom of peak 433 m/z and calculated peaks for species $[Ni(4DHBP)_2$-H] and $[Ni(4DHBP)_2]$.

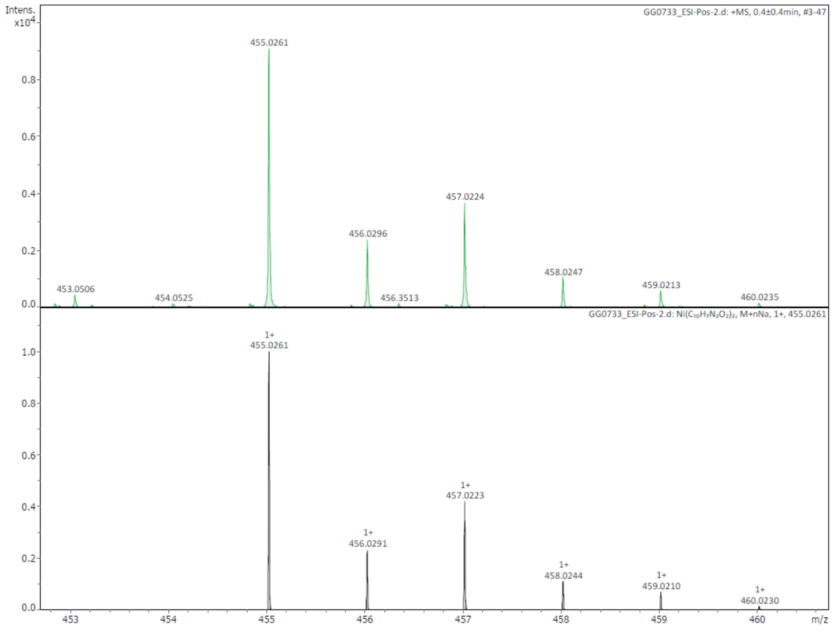

Figure S11: ESI–MS spectrum in H_2O of the reaction mixture for the synthesis of [Ni(4DHBP)(acac)] and $[Ni(4DHBP)_2]$, zoom of peak 433 m/z and calculated peaks for species $[Ni(4DHBP)_2]$Na.

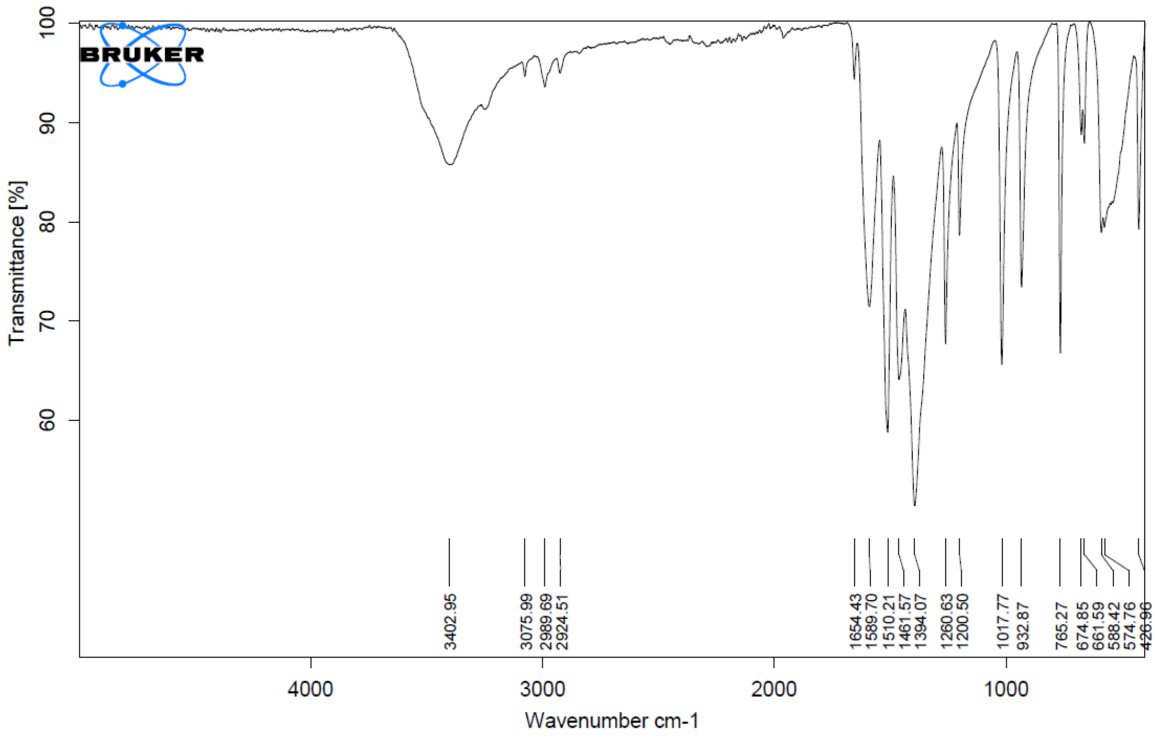

Figure S12: ATR–IR spectrum of [Ni(acac)$_2$].

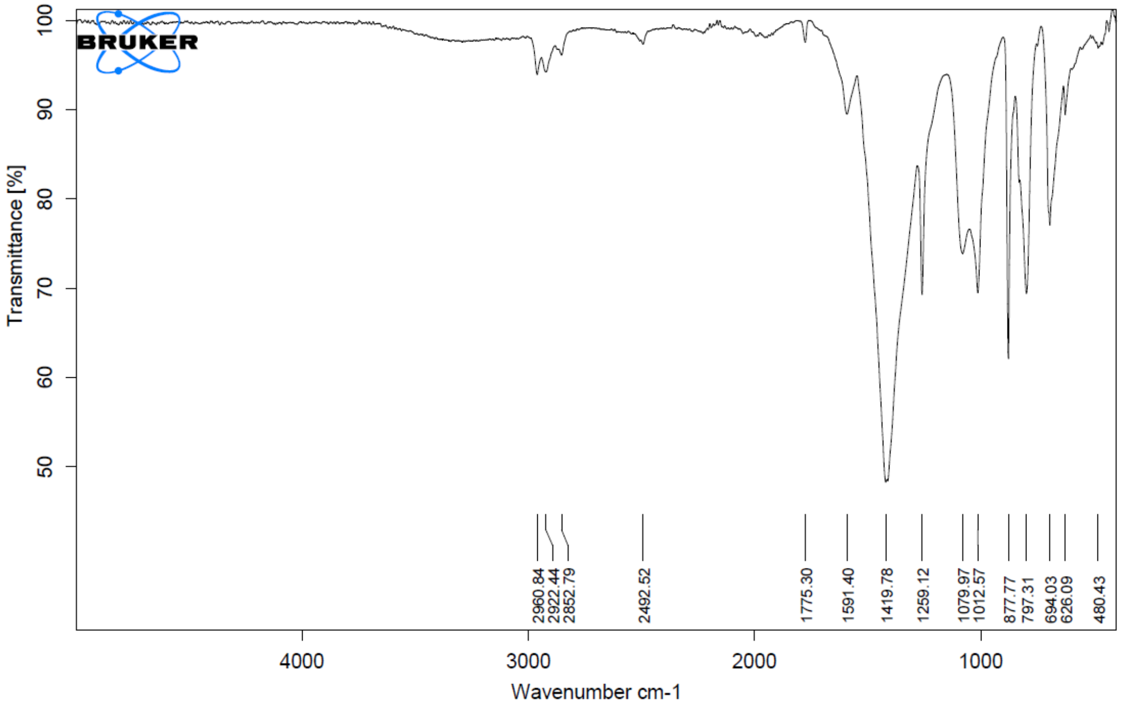

Figure S13: ATR–IR spectrum of [Ni(4DHBP)$_2$].

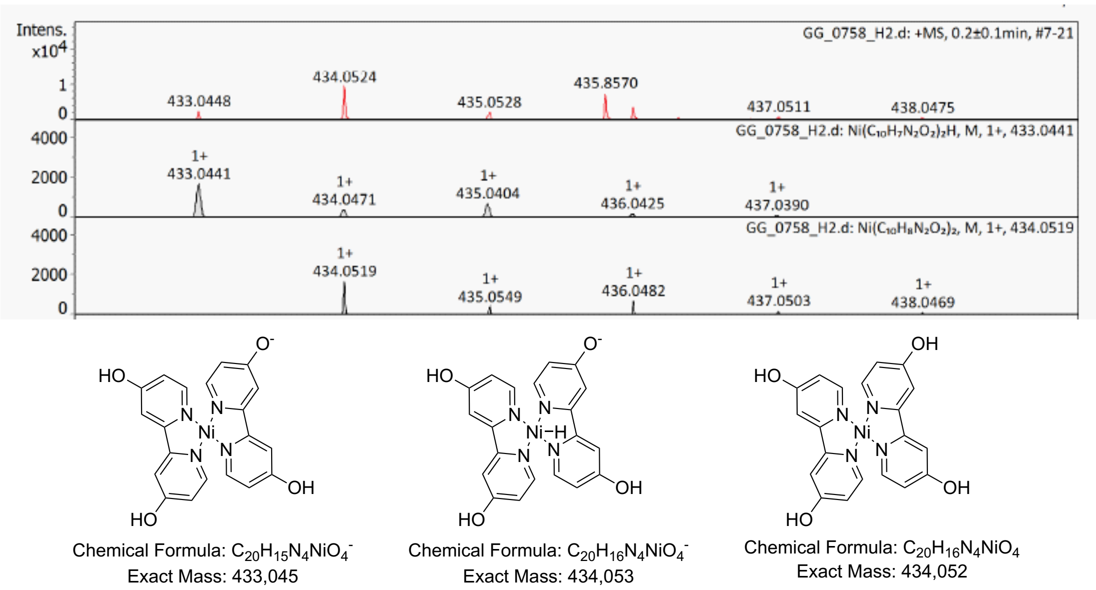

Figure S14: ESI–MS spectrum in H_2O of the hydrogenation reaction mixture, zoom at 435 m/z region and simulated mass spectra of $[Ni(4DHBP)_2\text{-}H]^+$, $[Ni(4DHBP)_2]$ and $[NiH(4DHBP)\text{-}H]^+$.

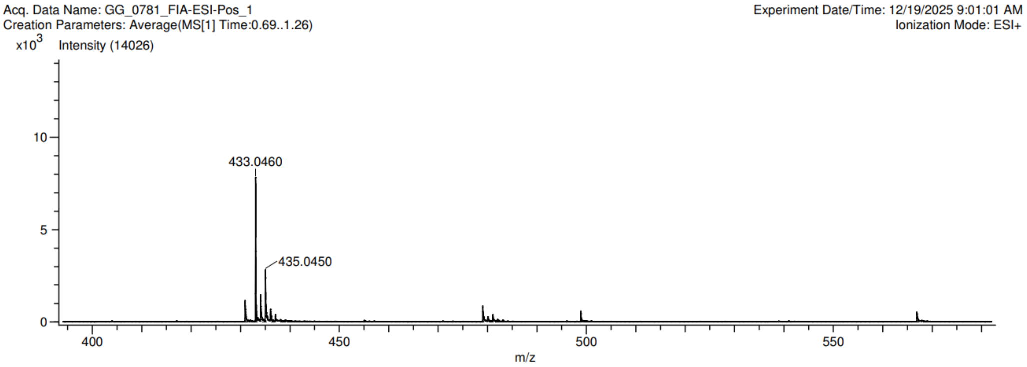

Figure S15: ESI–MS spectrum of the reaction mixture for the synthesis of $[Ni(4DHBP)_2(OOCH)]$, zoom on 433 m/z peak, corresponding to $[Ni(4DHBP)_2\text{-}H]^+$ (see Figure S14).

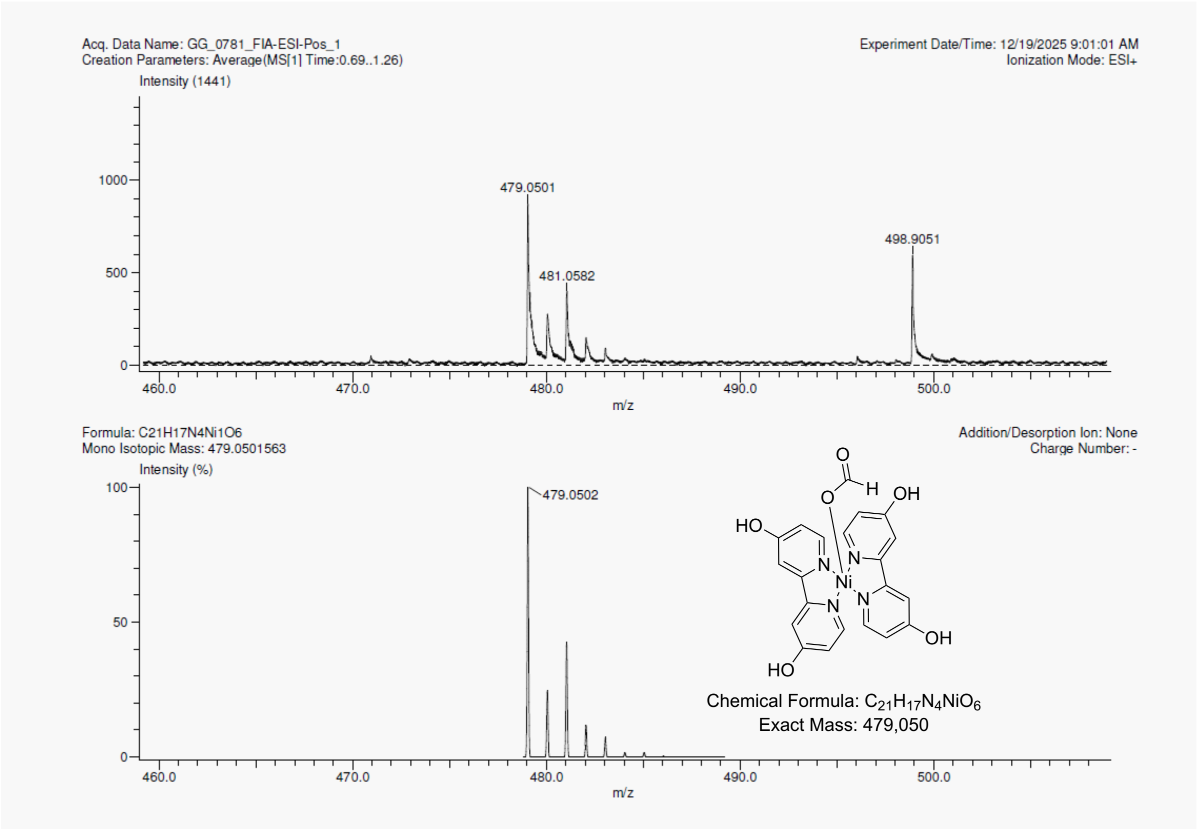

Figure S16: ESI–MS spectrum in H_2O of the reaction mixture for the synthesis of [Ni(4DHBP)$_2$(OOCH)].

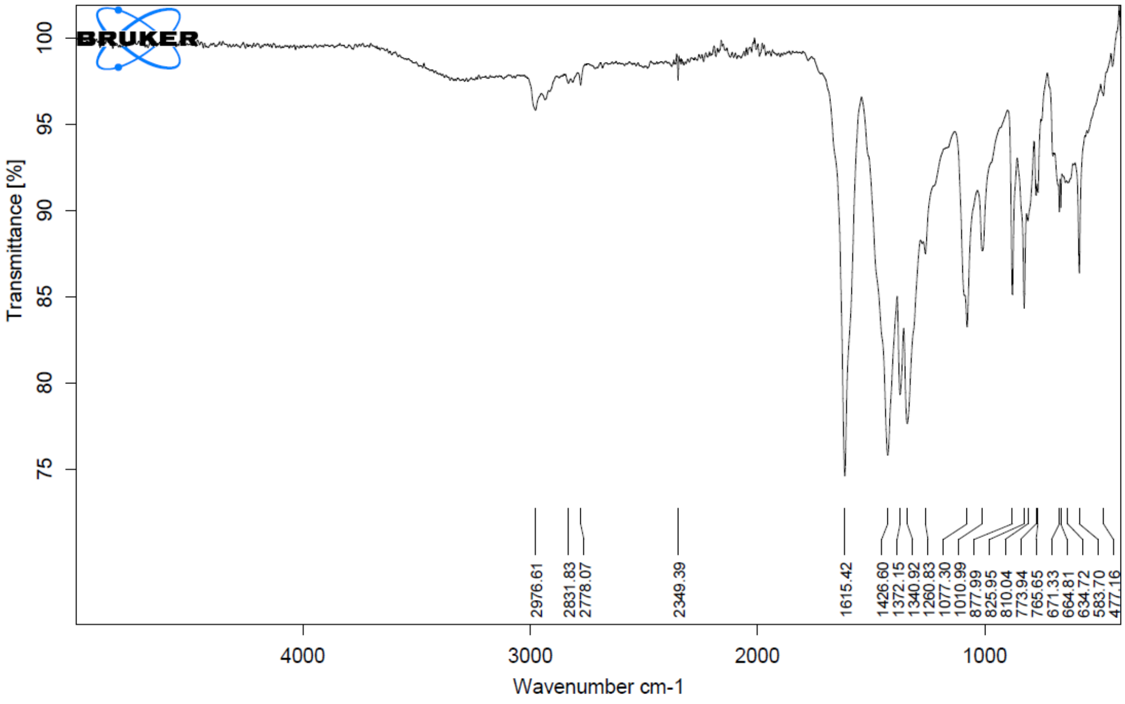

Figure S17: ATR–IR spectrum of [Ni(4DHBP)$_2$(OOCH)].

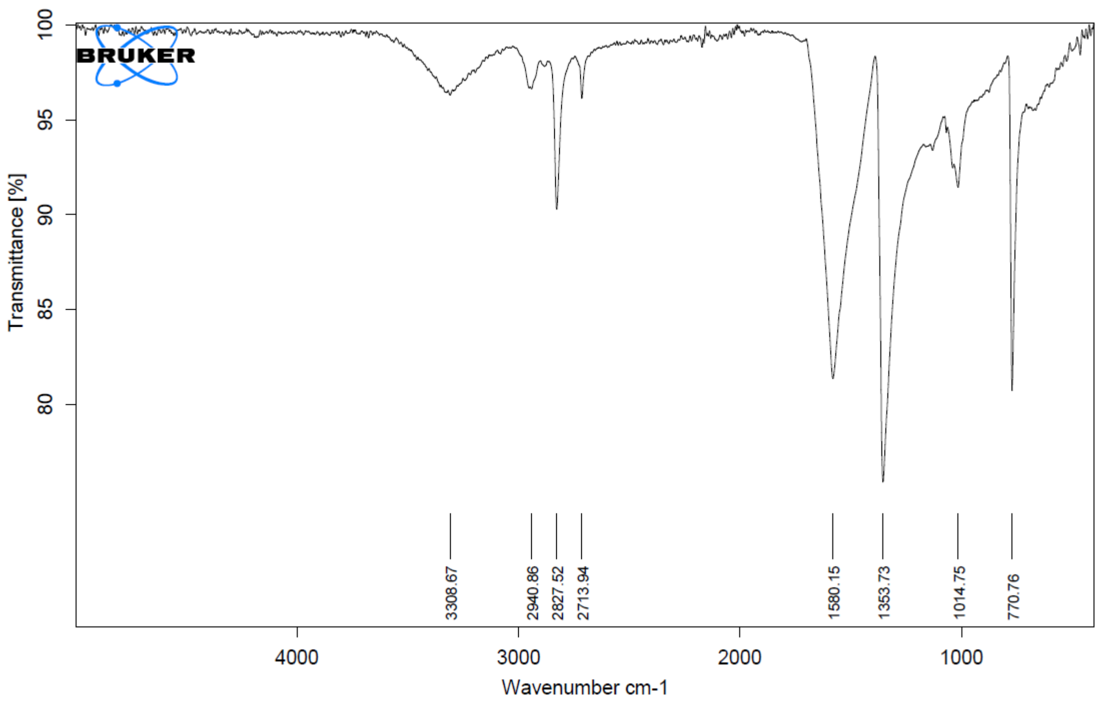

Figure S18: ATR–IR spectrum of HCOONa.

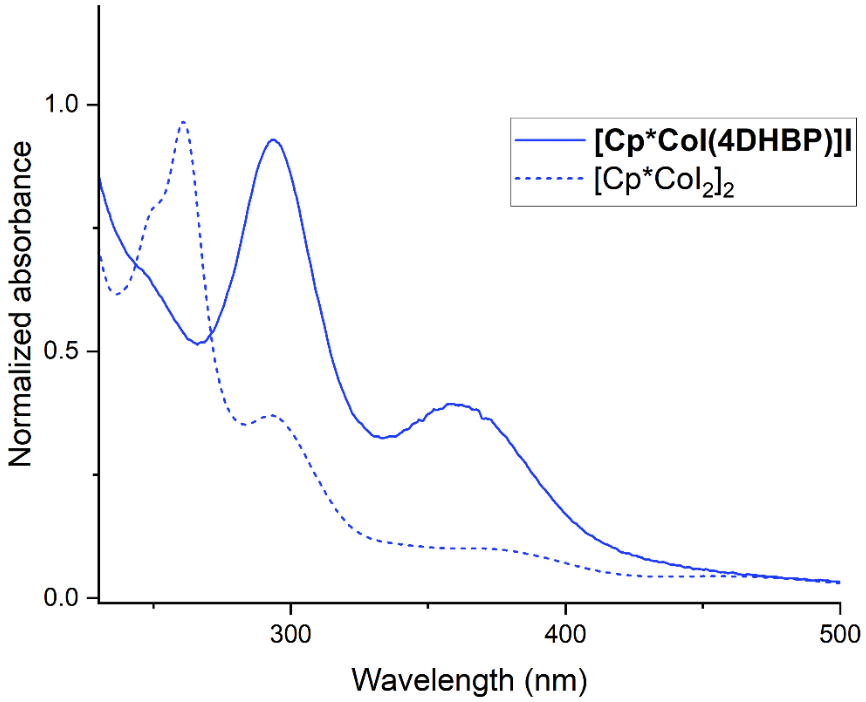

*Figure S19: UV/Vis spectrum of [Cp*CoI(4DHBP)]I (blue line) and [Cp*CoI$_2$]$_2$ (blue dotted line) in DCM.*

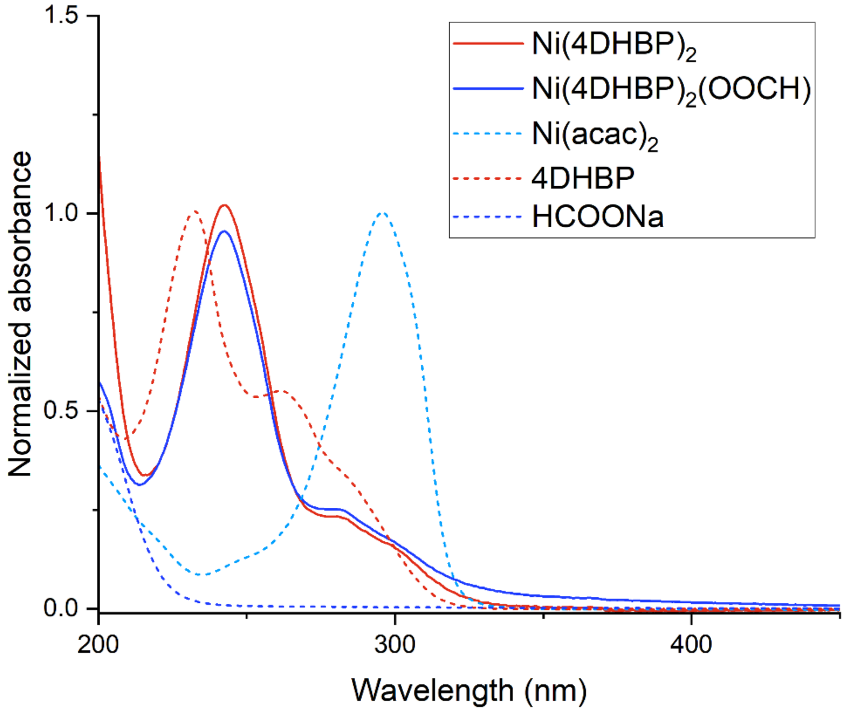

Figure S20: UV/Vis spectrum of [Ni(4DHBP)$_2$] (red line), [Ni(4DHBP)$_2$(OOCH)] (dark blue line), [Ni(acac)$_2$] (dotted light blue line), 4DHBP (dotted orange line) and HCOONa (dotted dark blue line) in H_2O.

SV. Crystallographic data

*Table S5: Crystallographic data and structure refinement details for Cp*Co(4DHBP)I*

**Compound**	**Cp*Co(4DHBP)I**
**CCDC number**	2546192
**Empirical formula**	$C_{21}H_{25}Cl_2CoIN_2O_2$
**Formula weight**	594.16
**Temperature [K]**	100(2)
**Crystal system**	orthorhombic
**Space group (number)**	$Pnna$ (52)
a **[Å]**	14.0589(4)
b **[Å]**	35.3601(9)
c **[Å]**	16.9765(5)
**α [°]**	90
**β [°]**	90
**γ [°]**	90
**Volume [Å^3]**	8439.4(4)
Z	16
**ρ_{calc} [gcm^{-3}]**	1.871
**μ [mm^{-1}]**	2.551
***F*(000)**	4720
**Crystal size [mm^3]**	0.364 × 0.048 × 0.035
**Crystal colour**	violet
**Crystal shape**	needle

**Radiation**	MoK_α (λ=0.71073 Å)
**2θ range [°]**	8.17 to 50.05 (0.84 Å)
**Index ranges**	$-16 \le h \le 16$, $-42 \le k \le 42$, $-20 \le l \le 20$
**Reflections collected**	179165
**Independent reflections**	7427, R_{int} = 0.1012, R_{sigma} = 0.0259
**Completeness to θ = 25.027°**	99.5 %
**Data / Restraints / Parameters**	7427/526/487
**Goodness-of-fit on F^2**	1.066
**Final R indexes [$I \ge 2\sigma(I)$]**	R_1 = 0.0345, wR_2 = 0.0847
**Final R indexes [all data]**	R_1 = 0.0502, wR_2 = 0.0938
**Largest peak/hole [eÅ^{-3}]**	1.67/-1.49
